# Development of Dipeptide *N*–acetyl–L–cysteine Loaded Nanostructured Carriers Based on Inorganic Layered Hydroxides

**DOI:** 10.3390/pharmaceutics15030955

**Published:** 2023-03-15

**Authors:** Denise Eulálio, Mariana Pires Figueiredo, Christine Taviot-Gueho, Fabrice Leroux, Cristina Helena dos Reis Serra, Dalva Lúcia Araújo de Faria, Vera Regina Leopoldo Constantino

**Affiliations:** 1 Departamento de Química Fundamental, Instituto de Química, Universidade de São Paulo—USP, São Paulo 05508-000, SP, Brazil; 2 Institut de Chimie de Clermont-Ferrand, Université Clermont Auvergne, BP 10448, F-63000 Clermont-Ferrand, France; 3 Centre National de la Recherche Scientifique (CNRS), UMR 6296, Institut de Chimie de Clermont-Ferrand (ICCF), F-63178 Aubiere, France; 4 Departamento de Farmácia, Faculdade de Ciências Farmacêuticas, Universidade de São Paulo—USP, São Paulo 05508-000, SP, Brazil

**Keywords:** layered double hydroxides, hydrotalcite-like compounds, intercalation compounds, layered materials, acetylcysteine, vibrational spectroscopy, in vitro drug release

## Abstract

*N*–acetyl–L–cysteine (NAC), a derivative of the L–cysteine amino acid, presents antioxidant and mucolytic properties of pharmaceutical interest. This work reports the preparation of organic-inorganic nanophases aiming for the development of drug delivery systems based on NAC intercalation into layered double hydroxides (LDH) of zinc–aluminum (Zn_2_Al–NAC) and magnesium–aluminum (Mg_2_Al–NAC) compositions. A detailed characterization of the synthesized hybrid materials was performed, including X-ray diffraction (XRD) and pair distribution function (PDF) analysis, infrared and Raman spectroscopies, solid-state ^13^carbon and ^27^aluminum nuclear magnetic resonance (NMR), simultaneous thermogravimetric and differential scanning calorimetry coupled to mass spectrometry (TG/DSC–MS), scanning electron microscopy (SEM), and elemental chemical analysis to assess both chemical composition and structure of the samples. The experimental conditions allowed to isolate Zn_2_Al–NAC nanomaterial with good crystallinity and a loading capacity of 27.3 (m/m)%. On the other hand, NAC intercalation was not successful into Mg_2_Al–LDH, being oxidized instead. In vitro drug delivery kinetic studies were performed using cylindrical tablets of Zn_2_Al–NAC in a simulated physiological solution (extracellular matrix) to investigate the release profile. After 96 h, the tablet was analyzed by micro-Raman spectroscopy. NAC was replaced by anions such as hydrogen phosphate by a slow diffusion-controlled ion exchange process. Zn_2_Al–NAC fulfil basic requirements to be employed as a drug delivery system with a defined microscopic structure, appreciable loading capacity, and allowing a controlled release of NAC.

## 1. Introduction

**Figure 1:**
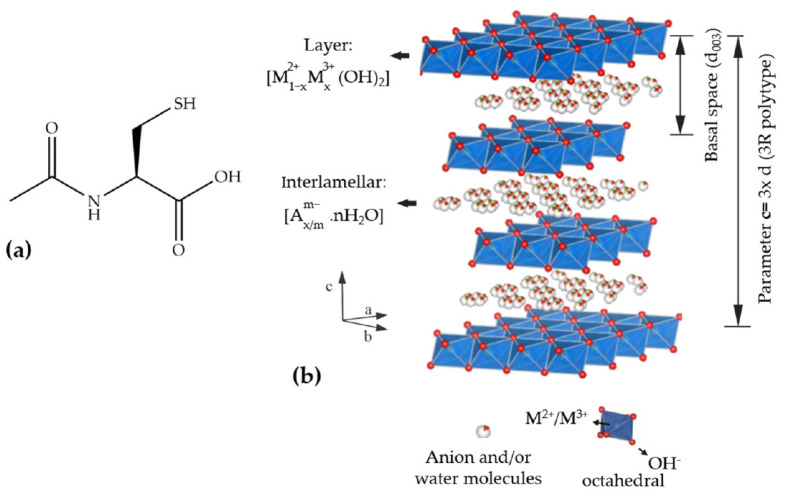
Schematic structural representation of (**a**) *N*–acetyl–L–cysteine and (**b**) LDH (ICSD number 91155) [30] obtained using the software Vesta (version 3).

*N*–acetyl–L–cysteine (NAC) or 2-acetamido-3-sulfanylpropanoic acid, with the chemical structure is shown in Figure 1a, is a derivative of the amino acid L–cysteine that has been used in therapeutic practices for several decades. In recent years, NAC has shown numerous biological properties, which have been studied by in vitro and in vivo preclinical trials, along with clinical data [1]. Firstly, it was used as a mucolytic agent in a study developed by Hurst et al. [2] in patients with cystic fibrosis showing that NAC can break disulphide bonds of mucus. Years later, the potential of NAC for the treatment of acetaminophen poisoning, paracetamol overdose [3], paracetamol-induced acute kidney injury [4], and to decrease hepatic injury [5] was reported. NAC has effective antioxidant properties and, consequently, a strong capability to be used in treatments related to the generation of free radicals and as a food supplement. This drug has therapeutic potential for the treatment of other diseases such as Alzheimer’s [6], type-2 diabetes [7], type-1 diabetes [8], gestational diabetes mellitus [9], diseases related to psychiatric disorders [10], neurodegenerative diseases [11], inhibition and induced death of lung cancer cells [12], HIV-associated tuberculosis [13], and bone regeneration approaches [14,15]. NAC is considered helpful in the nutraceutical control of RNA viruses such as influenza and coronavirus and has been investigated as an adjuvant to the prevention and therapy of COVID-19 [16,17,18] and post-COVID-19 pulmonary fibrosis [19]. As a source of thiol groups (-SH), NAC is an excellent precursor for the biosynthesis of the tripeptide γ–glutamyl–L–cysteinyl-glycine (GSH, glutathione) inside cells, increasing the levels of this peptide which acts in the elimination of free radicals [7,20,21,22,23]. Although NAC oral bioavailability is higher than that of GSH, it is very low (4 to 10%), possibly due to the fact that plasma is a pro-oxidant medium, thus favoring the oxidation of NAC to symmetrical (NAC-NAC) or asymmetric disulphides (Cyst-NAC) [21,24,25]. NAC can be administered orally, intravenously, or by inhalation, and its terminal half-life (t_1/2_) is approximately 6 h [26,27]. When administered orally, the maximum plasma concentration (C_max_) is reached after 1–2 h [27]. NAC is classified as a biopharmaceutics classification system (BCS) I drug and shows high solubility of 100 mg mL^−1^ [28,29].

The confinement of NAC species into inorganic nanoparticles as layered double hydroxides (LDH) can be an interesting approach because the architecture of these materials has improved the physical–chemical stability, biocompatibility, and bioavailability of several bioactive species intercalated between the layers and also possess release properties, as reported in several review articles [31,32,33]. LDH are 3D structures formed by 2D layers [34], as shown in Figure 1b. LDH layers are held by electrostatic interactions and hydrogen bonds; each layer is formed by hydroxide ions coordinated to bivalent and trivalent cations in an octahedral geometry. The [M(OH)_6_] units are joined by the edges forming a positively charged layer, which grows along the *ab* plan [35]. The general formula [M_1−x_^2+^M_x_^3+^(OH)_2_]A^m−^_x/m_·*n*H_2_O is associated with LDH, wherein M^2+^ and M^3+^ denote bivalent and trivalent cations, respectively, and A^m−^ represents an intercalated anion with negative charge *m*. LDH composition is abbreviated in this work as M^2+^_R_M^3+^-A, where R is the M^2+^/M^3+^ cation molar ratio. By varying the M^2+^ ions (Zn, Mg, Ni, Cu, Co, Mn, or Fe, for instance), the M^3+^ ions (Al, Fe, Cr, Co, Mn, Ga, or Ni, for example), and the intercalated anion, a wide variety of these layered matrices can be synthesized in a controlled way [36]. 

The LDH composition, size, morphology, surface charge, and functionality can be tuned to load active principles of biomedical interest for therapeutic use, diagnosis, or theranostic applications [37]. The hydroxide layers give the pH-responsive property to this class of inorganic materials, allowing the bioactive species release not only by anion exchange reaction but also by the carrier solubilization in acidic sites originating from inflammatory processes or malignant diseases, for instance. The LDH is not an inert material in the live organism; it can be decomposed by slow acid-base reaction and/or complexation reaction with biomolecules. The nature of the chemical elements that comprise the layers is also very relevant because LDH solubility depends on the cations as well as the body’s response to such delivered cations. Actually, LDH shows intrinsic biological properties related to M^n+^ ions that can modulate the repair of injured tissues [38], cause apoptosis of cancer cells by regulation of gene expression [39], present immunomodulating activity [40] or osteogenic differentiation [41], or promote the induction of neovascularization and angiogenesis [42,43], among other activities. 

The intercalated species studied cover a wide range of properties such as anti-inflammatory, antibiotic, antitumor, anticoagulant, antimicrobial, antidepressant, and antioxidant properties [44,45]. We have worked on the development of LDH host matrices for use as carriers of anionic species derived, for instance, from ibuprofen [46], sulindac [47], mefenamic acid [48], coumaric acid [49,50], pravastatin [51], naproxen [43], norbixin [52], and ciprofloxacin [53]. For some LDH materials, in vivo tests for phases intercalated with chloride ions (Mg_2_Al–Cl and Zn_2_Al–Cl [42], Mg_4_FeAl–Cl and Zn_4_FeAl–Cl [43]), or coumarate (Mg_2_Al–Cou and Zn_2_Al–Cou) [49] were performed to assess their biocompatibility by intramuscular implants, while in vitro assays of intercalated sulindac (Mg_2_Al–Sul and Zn_2_Al–Sul) [47] and mefenamate (Mg_2_Al–Mef) [48] materials were also studied. Furthermore, the release of sulindac from Zn_2_Al-Sul was evaluated in vivo by Raman spectroscopy from intramuscular implants [54]. The amazing results obtained in these in vivo tests concerning LDH composed by Mg, Zn, Fe, and Al ions open the possibility to evaluate them for implantable delivery systems because the formation of a fibrotic capsule was not observed. When the LDH intercalated with active species were prepared as nanofibrous membranes by poly(lactic acid) electrospun and electrosprayed LDH at the same time, the drug release extended for up to 66 days [55]. Hence, LDH is a potential material for the development of implantable multi-functional composites [56]. 

To the best of our knowledge, the intercalation of NAC into LDH was never reported before. Some studies showed the intercalation of L–cysteine, an analogue amino acid. Wei et al. [57] studied the intercalation of L–cysteine in matrices of Mg_2_Al composition as a reactor for the transformation of chemical substances. The authors observed that L–cysteine was oxidized within the LDH environment. Stimpfling et al. [58] intercalated L–cysteine into LDH of composition LiAl_2_, Mg_2_Al, and MgZnAl to assess the ability of the synthesized materials to prevent corrosion of the AA2024 aluminum alloy. Silva [59] explored the intercalation of L–cysteine into LDH matrices of composition Mg_3_Al and Mg_3_Fe to investigate how LDH type-structure minerals present on the planet in ancient times could have interacted with species of biological interest.

The main aim of this work was to investigate experimental parameters to obtain nanodelivery drug systems (DDS) based on LDH (with compositions Zn_2_Al and Mg_2_Al) intercalated with the dipeptide NAC as well as to perform their detailed physicochemical characterization and the drug release kinetic models. Well structurally organized Zn_2_Al–NAC hybrid material was isolated with high NAC loading capacity while, in the presence of the Mg_2_Al–LDH composition, the partial oxidation of NAC was observed, i.e., its chemical integrity was not preserved. In vitro drug delivery tests were performed in two conditions in which the release medium was constantly agitated in a dissolution tester (basket method, here abbreviated S1) or not agitated (abbreviated S2) during the kinetic studies. Agitation of the release medium can impact the diffusion of water and ionic species into a tablet and the successive drug release. Such a simple method is also known as sample and separate (SS) method. Powdered samples were made in the form of cylindrical tablets, avoiding the presence of nanoparticles in the drug analysis medium, which cannot be sedimented by centrifugation or retained by membrane filtration; the simulated physiological solution used in the released assays aimed to mimic the extracellular matrix solution, as reported in the literature [60], considering the previously mentioned works about in vivo implantation of tablets to evaluate the DDS biocompatibility.

## 2. Materials and Methods

### 2.1. General

*N*–acetyl–L–cysteine (C_5_H_9_NO_3_S), zinc chloride (ZnCl_2_), magnesium chloride hexahydrate (MgCl_2_·6H_2_O), and aluminum chloride hexahydrate (AlCl_3_·6H_2_O) were supplied by Sigma-Aldrich (St. Louis, MO, USA), while sodium hydroxide (NaOH) was provided by Merck (Darmstadt, Germany). NaCl and MgCl_2_∙6H_2_O were provided by Merck (Darmstadt, Germany). NaHCO_3_, KCl, K_2_HPO_4_, HCl, CaCl_2_, Na_2_SO_4,_ and Tris hydroxymethyl aminomethane were provided by Synth (Diadema, SP, Brasil). All chemicals were used without further purification.

### 2.2. Synthesis of Layered Double Hydroxides 

M_2_Al–NAC (M = Zn or Mg) samples were prepared by the co-precipitation method at constant pH. A 0.1 mol L^−1^ solution of metal cations with M/Al molar ratio equal to 2 (14.0 mmol of M^2+^ and 7.0 mmol of Al^3+^) was prepared employing MgCl_2_·6H_2_O and ZnCl_2_ salts dissolved with deionized water (from Millipore, model Direct-Q 8 UV Smart (Jaffrey, EUA). Then, the aqueous solution was slowly added under stirring, using a magnetic stirrer (model 752A by Fisaton, Perdizes, Brazil), to a 0.1 mol L^−1^ solution of NAC (NAC/Al^3+^ molar ratio equal 1) at room temperature or at 55 °C and under nitrogen gas flow. The pH value was kept constant at 7.5 for Zn_2_Al–LDH and at 9.5 for Mg_2_Al–LDH by the addition of a 0.2 mol L^−1^ solution of NaOH. After the complete addition of the metal cations, the suspension formed from metal hydroxide co-precipitation was kept under stirring for 1 h under nitrogen gas flow. The solid materials were separated by centrifugation using an equipment model Z36 HK by Hermle (Wehingen, Germany) for 10 min at 10 krpm, washed several times with deionized water, washed a final time with ethanol, and dried under reduced pressure (vacuum pump, Fanem LTDA, São Paulo, Brazil). The samples synthesized at room temperature or at 55 °C were abbreviated respectively as M_2_Al–NAC or M_2_Al–NAC55. Another condition was evaluated for the preparation of the zinc hybrid material keeping the same experimental conditions but using an NAC/Al^3+^ molar ratio equal to 1.3 (sample named Zn_2_Al–1.3NAC55).

The same experimental conditions mentioned to synthesize LDH–NAC phases were applied to obtain LDH samples intercalated with chloride ions: the solution of metal cations was under stirring slowly added to 50 mL of deionized water. According to the results of metal and water analyses, the proposed composition for Zn_2_Al–Cl55 and Mg_2_Al–Cl was, respectively, [Zn_2.0_Al(OH)_6.0_]Cl_0.90_(CO_3_)_0.05_·1.42H_2_O and [Mg_1.8_Al(OH)_5.6_]Cl_0.95_(CO_3_)_0.025_·1.83H_2_O. 

### 2.3. Preparation of NAC Salts in the Sodium Form

First, 0.2 mol L^−1^ solution of NaOH was added to 0.1 mol L^−1^ solution of NAC under vigorous stirring and N_2_ atmosphere at room temperature. The NaOH solution was added to NAC solution up to reach the pH values 7.5, 8.5, 9.5, or 11. Posteriorly, the four solutions were frozen, freeze-dried (in a Thermo Savant Modulyo D equipment, Madrid, Spain), and kept under reduced pressure. The isolated NAC salt samples were abbreviated NAC-pH = X, where X is the pH value of the freeze-dried solution.

### 2.4. In Vitro NAC Release Kinetics Experiments

Briefly, 40 mg of powdered Zn_2_Al–NAC55 samples containing 11 mg of NAC were compressed into tablets of 5 mm in diameter and 1 mm in thickness using a manual hydraulic tablet press employing 0.25 tons for 5 min. The release of NAC from the tablets was performed in simulated body fluid (SBF) at pH 7.4, prepared by dissolving NaCl (137.5 mmol), NaHCO_3_ (4.2 mmol), KCl (3.0 mmol), K_2_HPO_4_ (1.0 mmol), MgCl_2_∙6H_2_O (1.6 mmol), HCl (39 mL of 1 mol L^−1^ solution), CaCl_2_ (2.6 mmol), Na_2_SO_4_ (0.5 mmol), and tris hydroxymethyl aminomethane (50.5 mmol) in 1 L of deionized water, according to the literature [60]. The drug release experiments using the method with agitation (S1 method) were achieved in a dissolution instrument using the USP Apparatus 1, in which the basket rotated at 50 rpm [61]. Zn_2_Al–NAC55 tablets were immersed in 50 mL of SBF solution at 37 °C and stirred for 96 h. At predetermined intervals, 3 mL of SBF solution was removed from the apparatus and replenished with an equal volume of fresh SBF medium. The experiments were made in triplicate. 

In the release tests performed at static conditions without agitation (S2 method), the Zn_2_Al–NAC55 tablets were placed in Eppendorf microtubes to which were added 2 mL of SBF solution at 37 °C. At predetermined time intervals, 2 mL of the release medium was removed and replaced by equivalent volumes of fresh SBF medium. The release assays were performed in triplicate. The NAC concentration in both release conditions (S1 and S2 static methods) was determined by UV-visible absorption spectrophotometry at the wavelength of maximum absorbance (λ_max_) equal to 208 nm, while the metal cations amount in the release medium was quantified by inductively coupled plasma optical emission spectrometry (ICP OES). The stratigraphic analyses of the Zn_2_Al–NAC55 tablets after the release tests were conducted by micro-Raman spectroscopy.

The suitable mathematical models [62,63,64,65] employed to verify the NAC release kinetics from Zn_2_Al–NAC55 tablets are indicated in Table 1. The statistical analysis of variance (ANOVA) considered a significance level of 0.05 [65]. 

### 2.5. Equipment

X-ray diffraction (XRD) patterns of powdered samples were recorded in a D8 Discover Bruker diffractometer (Karlsruhe, Germany) using Cu Kα radiation (1.5406 Å), 40 kV, 30 mA and Lynxeye detector (192 segments). Data were collected in the (2θ) 3.0–70° range and using a scan step of 0.02° (2θ)/0.5 s. 

The atomic PDF was obtained from X-ray total scattering data collected on a PANalytical Empyrean diffractometer equipped with a solid-state GaliPIX3D detector, a focusing X-ray multilayer mirror, and an Ag anticathode (Kα_1_ = 0.5594 Å, Kα_2_ = 0.5638 Å). Powder samples were mounted in glass capillaries of 0.7 mm diameter. An empty capillary of the same type was measured in the same way for background subtraction. Data were recorded over the range 1 < 2θ < 145°, which corresponds to an accessible maximum value for the scattering vector Q_max_ of 21.4 Å^−1^**.**

Scanning electron microscopy (SEM) images were obtained in an electron microscope Jeol-JSM 6610LV equipment (Tokyo, Japan) with 7 kV voltage at secondary electrons (SE) mode. Sampling was performed by spreading the powder directly on the carbon tape and posteriorly coating it with a gold film using a sputter Denton Vacuum, model DESK V (Moorestown, NJ, USA). 

Fourier transform infrared (FTIR) spectra of powdered samples were recorded in a Bruker spectrophotometer (Ettlingen, Germany), Alpha model, in the attenuated total reflectance (ATR) accessory (Platinum) with diamond crystal, in the 400–4000 cm^−1^ range, with a spectral resolution of 4 cm^−1^; 256 scans were co-added.

Fourier transform Raman (FT–Raman) spectra of powdered samples were recorded in an FT–Raman Bruker RFS-100/S device (Ettlingen, Germany) using 1064 nm exciting radiation from Nd:YAG laser Coherent Compass 1064–500 N (Lübeck, Germany), liquid N_2_ cooled Ge detector, laser power of 140 mW at the sample, 1024 scans, a spectral resolution of 4 cm^−1^, and in the 100–3500 cm^−1^ range. 

Raman spectra of Zn_2_Al–NAC55 tablets after the drug delivery experiments were recorded on a Renishaw in Via Reflex microscope (Wotton-under-Edge, Gloucestershire, UK), equipped with a thermoelectrically cooled CCD camera (Renishaw, 600 × 400 pixels) coupled to a Leica microscope model DM2500M; the laser line at 785 nm (diode laser, Renishaw) was focused on the sample by a Leica x50 objective (numerical aperture 0.75).

Thermogravimetric and differential scanning calorimetry coupled to mass spectrometry (TG/DSC–MS) curves were registered with a Netzsch device, model TGA/DSC 490 PC Luxx (Spectro Analytical Instruments GmbH, Selb, Germany), coupled to an Aëlos 403C mass spectrometer (Germany). Analysis was performed under a synthetic air flow of 50 mL min^−1^ employing alumina crucibles and a heating rate of 10 °C min^−1^.

Chemical analyses of Mg, Zn, and Al metals were performed in triplicate by inductively coupled plasma optical emission spectrometry (ICP AES) on a spectra Arcos spectrometer (Kleve, Germany) with axial plasma observation at the Central Analítica of the Instituto de Química of USP (CA–IQUSP). Samples were solubilized in a 4 mol L^−1^ solution of nitric acid and diluted before the measurements. 

Carbon, nitrogen, and hydrogen elemental analysis were recorded with a Perkin Elmer–CHN device (Waltham, MA, USA), model 2400, at CA–IQUSP.

Solid-state nuclear magnetic resonance spectroscopy (ss-NMR) spectra were recorded in a 300 Bruker Advance spectrometer (Rheinstetten, Germany). It employed magic-angle spinning (MAS) at 10 kHz using a 4 mm diameter size zirconia rotor. ^13^C (I = 1/2) spectra were recorded at 75.47 MHz by proton enhanced cross-polarization method (CP, contact time 1 ms, recycling time of 5 s) and referenced to the carbonyl of glycine calibrated at 176.03 ppm and 2000 to 10,000 scans. 

^27^Al (I = 5/2) spectra were recorded at 72.30 MHz, applying an accumulation of π/6 single-pulse, recycling time of 10 s, and calibration with a 1 mol L^−1^ aqueous solution of AlCl_3_ at 0 ppm.

Tablets of powdered samples were prepared using a manual hydraulic tableting press (Marconi MA 098, Wabash, IN, USA). NAP delivery experiments were conducted in a Pharma Test Dissolution Instrument type PTWS 610 (Hainburg, Germany). 

UV-VIS electronic spectra of NAC released in the kinect experiments were recorded on a Shimadzu UV-1650PC spectrophotometer (Kyoto, Japan) using quartz cuvette of 10 mm.

### 2.6. X-ray Diffraction Data Processing

The unit cell parameters for Zn_2_Al–NAC55 were determined from the full pattern matching refinement (Le Bail method) of X-ray diffraction patterns assuming R-3m space group and using Fullprof suite program [66]. The Patterson map was calculated using GFourier program [67] by employing the observed structure factors Fobs extracted from profile matching and plotted in the form of contour plots summed from 0 to 1 along the a/b-axis. The program HighScore Plus software provided by PANalytical Corporation was used for converting total X-ray scattering data to an atomic pair distribution function PDF of G(r). Fourier transforms of the reduced structure functions S(Q) were truncated at 21 Å^−1^. The bulk chemical composition [Zn_2.04_Al(OH)_6.08_](C_5_H_7_NO_3_S)_0.5_]·1.1H_2_O was used for the normalization of S(Q).

### 2.7. Simulation of NAC Vibrational Spectrum

Spartan 18 (Wavefunction Inc., Irvine, CA, USA) version 1.2.Ø [68] was employed in geometry optimization and vibrational frequencies calculation. Equilibrium geometry was calculated using the PM3 semi-empirical method [69] with molecular mechanics amide correction to obtain vibrational frequencies. Density functional theory (DFT) calculation was performed using the B3LYP density functional [70] and 6–31 G* as basis set. 

## 3. Results and Discussion

### 3.1. XRD, SEM, and Vibrational Spectroscopic Characterization of Zn_2_Al–NAC Sample

Preliminary experiments were conducted by varying the temperature of synthesis and the NAC/Al molar ratio to isolate single and well-crystallized phases of NAC intercalated into LDH. XRD patterns of Zn_2_Al–NAC and Zn_2_Al–NAC55 samples (see Supplementary Material, Figure S1) indicated that aging at 55 °C increased the crystallinity of the materials as evidenced by the better signal-to-noise ratio for (00ℓ) Bragg peaks when compared to the sample prepared at room temperature. Furthermore, the presence of additional harmonic basal peaks ((0012) and (0015) reflections) for the heated sample was also indicative of a better stacking of the layers. As seen in Figure S1, a molar excess of 30% of NAC did not improve its crystallinity, and a similar final composition was obtained for both samples with an NAC/Al molar ratio equal to 0.65 (Zn_2_Al-1.3NAC55) and 0.68 (Zn_2_Al–NAC.55). Therefore, the Zn_2_Al–NAC55 sample was selected to perform this study. 

**Figure 2:**
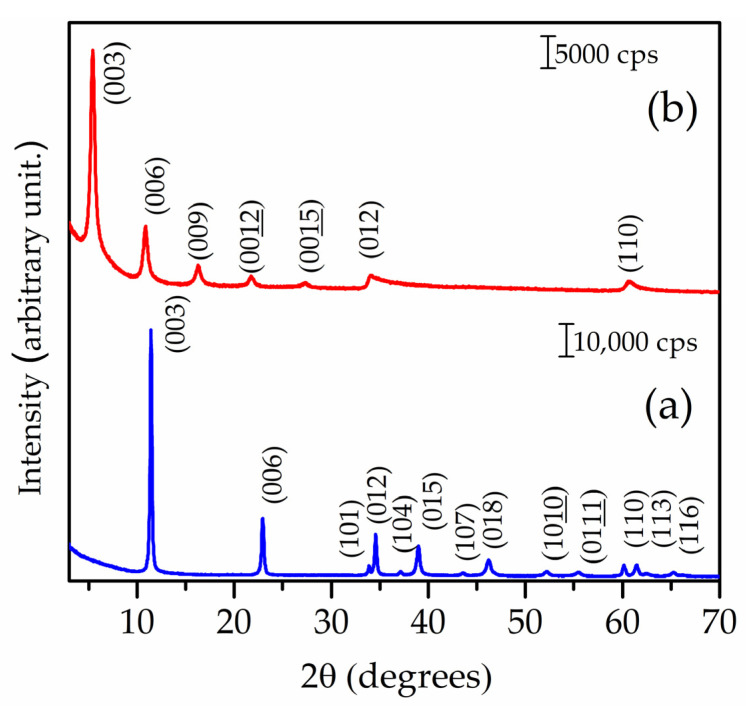
XRD patterns of (**a**) Zn_2_Al–Cl55 and (**b**) Zn_2_Al–NAC55 materials.

XRD patterns of Zn_2_Al–NAC55 and Zn_2_Al–Cl55 samples are characteristic of layered materials (Figure 2): the position of the 00ℓ reflections below about 2θ = 30° associated with the interlayer distances shows an increase in the basal spacing from 0.75 nm for Zn_2_Al–Cl to 1.63 nm for Zn_2_Al–NAC55, indicating the successful intercalation of the organic species. Furthermore, the shift of the (113) reflection to a lower angle almost coinciding with the (110) as expected with the increase of the interlayer distance was also in favor of the formation of a single LDH–NAC phase, i.e., the absence of LDH–Cl or LDH–CO_3_ phases. XRD peaks of NAC polymorphs, shown in Figure S2, were also not observed in the pattern of Zn_2_Al–NAC55, precluding the presence of crystalline-free NAC salt in the hybrid material.

**Figure 3:**
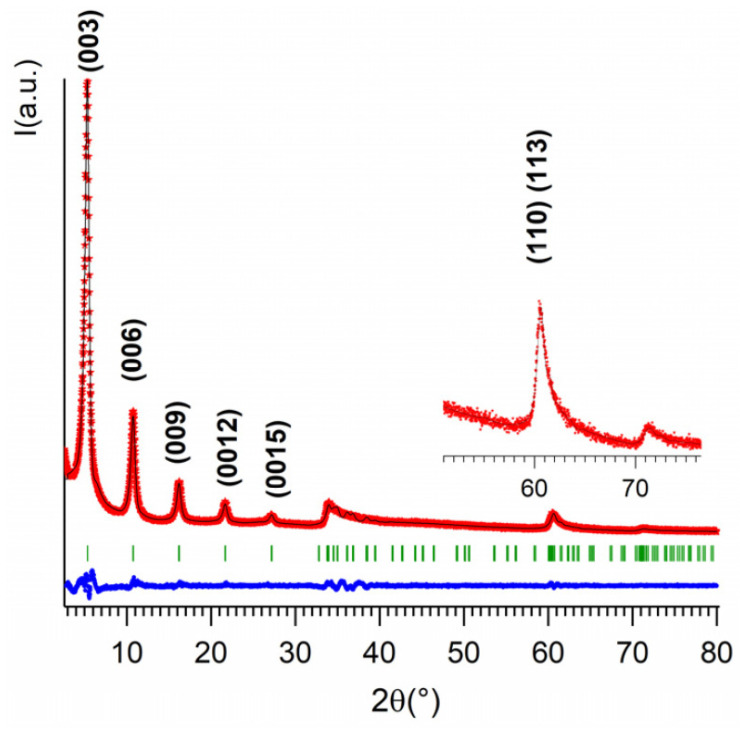
Results of the full pattern fitting of the X-ray diffraction pattern of Zn_2_Al–NAC55 using Le Bail method. Refinement in a hexagonal cell with R-3m space group: a = 3.066 Å, c = 49.144(3) Å. Reliability factors R_p_/R_wp_(%) = 12.0/11.5. Experimental XRD (red stars), calculated (black line), Bragg reflections (green ticks), and difference profile (blue line).

The good crystallinity of the Zn_2_Al–NAC55 sample, as well as the absence of crystalline impurities, allowed us to perform a whole diffraction pattern profile refinement (Figure 3). The obtained data were consistent with R-3m space group which is often reported for LDH materials [35]. An interlayer distance of d = 16.38 Å was deduced from the value of the c parameter of the hexagonal cell. Additionally, the value obtained for the cell parameter a = 3.066 Å indicated a Zn/Al molar ratio of 2.1 Å [71], i.e., a very close value to that one applied during the synthesis (a nominal Zn/Al value equal to 2).

**Figure 4:**
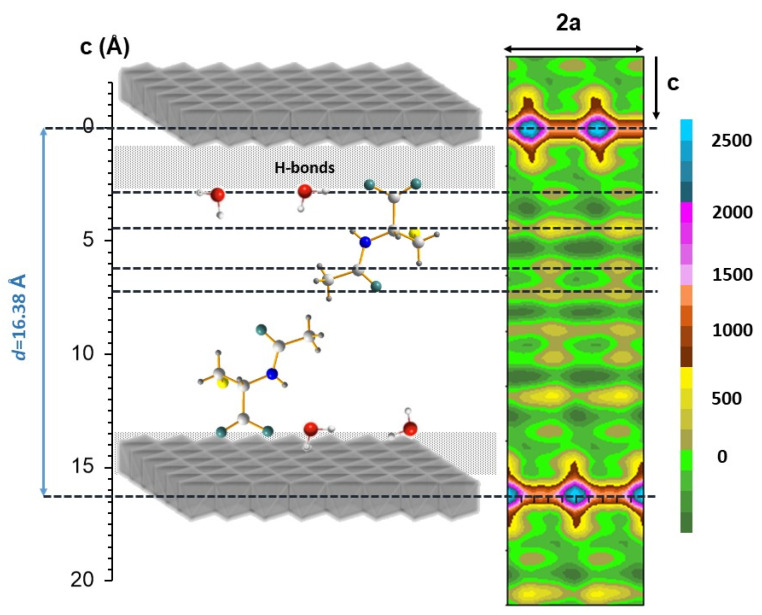
Structural model for Zn_2_Al–NAC55 deduced from the Patterson contoured map summed from 0 to 1 along the b-axis and calculated from the profile refinement of XRD data; the electron density scale given on the right is in arbitrary units.

The Patterson map was also calculated by considering all the integrated intensities extracted from the Le Bail fitting. The electron density in the interlayer space of Zn_2_Al–NAC55 sample is quite low (Figure 4) but nevertheless shows a distribution in different planes perpendicular to the c-stacking direction. The quasi-absence of electron density in the middle of the interlayer space confirms the bilayer arrangement. Carboxylate groups together with water molecules are likely to be located at the outer part of the interlayer space near the hydroxide layer at a distance around 3 Å from the centre of it, thus indicating the formation of rather strong hydrogen bonding with the OH groups of the layers. As one moves along the c-stacking direction, a pronounced local maximum detected at about 2 Å from the carboxylate groups may be attributed to the presence of heavy atoms such sulfur. Then, the amide group is observed.

**Figure 5:**
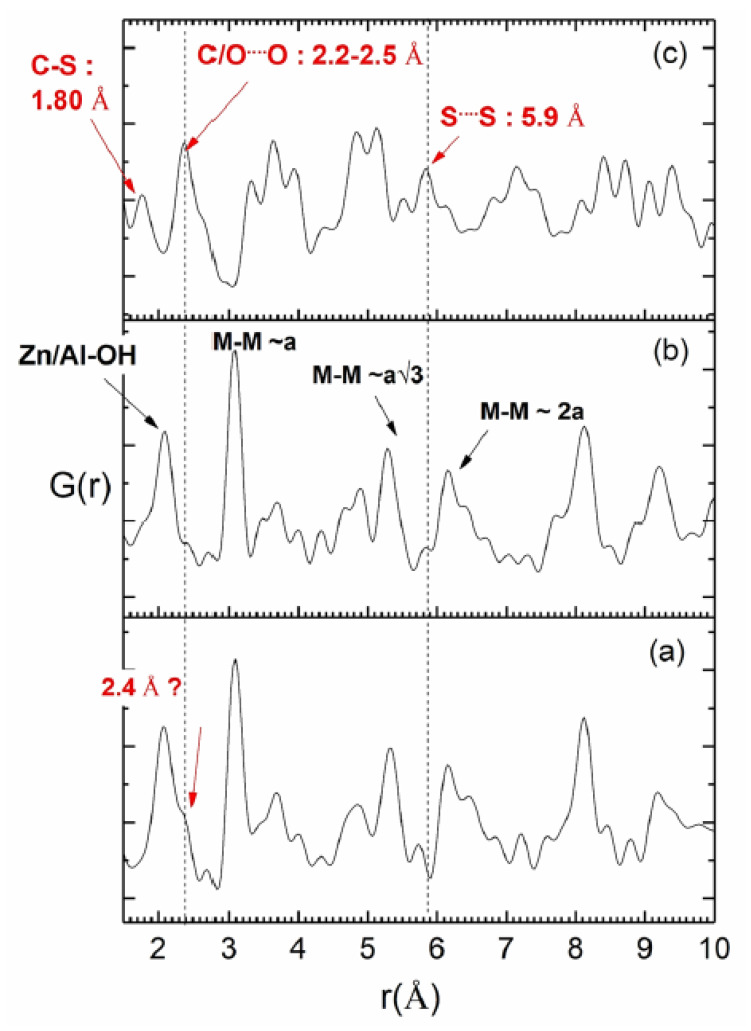
Experimental pair distribution functions PDF of (**a**) Zn_2_Al–NAC55 samples compared to (**b**) Zn_2_Al–Cl55 and (**c**) NAC as reference materials.

The PDF curves were extracted from total X-ray scattering data and, to facilitate interpretation, and the same analysis was performed on Zn_2_Al–Cl55 sample and NAC salt. As reported elsewhere [72], the first peak observed on the PDF for LDH materials refers to the hydroxide layers. Thus, in the present case, the first peak around 2.0 Å is due to the closest OH shell around Zn, Al atoms while the peaks observed at about 3.07 Å (a), 5.3 Å (√3a), and 6.2 Å (2a) are attributed to the M−M bond distances The other peaks are due to multiple pairs of atoms. Due to the high X-ray scattering power of the Zn atom, the PDF signal of [Zn_2_Al(OH)_6_] hydroxide layers is very intense, making it difficult to observe the signal from the interlayer space. The PDF curve of Zn_2_Al–NAC55 sample shows no change in the M-OH/M-M distances within the hydroxide layers. A shoulder around 2.4 Å is, however, noted on the first peak (Figure 5a), which is not observed in the PDF of Zn_2_Al–Cl55 (Figure 5b). The PDF curve of NAC (polymorph I) displays interatomic distances in this range (Figure 5c) corresponding to both intra and inter molecular distances as indicated by single-crystal XRD of NAC (Figure S3) [73]. Indeed, NAC molecules can interact by intermolecular and intramolecular hydrogen bonds, as, for instance, the intermolecular interactions between NH^•••^S (2.82 Å), CH^•••^OCOH (2.72 Å), and the intramolecular interaction between NH^•••^OCOH (2.26 Å). Calculations by DFT indicated that the more stable conformers for NAC (neutral compound) in a gas phase show main intramolecular bond lengths in the 2.24–2.32 Å range [74]. Hence, this additional distance observed at about 2.4 Å in Zn_2_Al–NAC55 may be attributed to the repetition of the drug array within the interlayer region. The interlayer space of LDH is a constrained environment and the distances among intercalated NAC anions can be distinct from those ones of the molecules in the crystal (Figure S3). Hence, the interatomic distance at 2.4 Å should involve a heavy atom such as sulfur because of its high X-ray scattering power compared to that of other NAC atoms.

**Figure 6:**
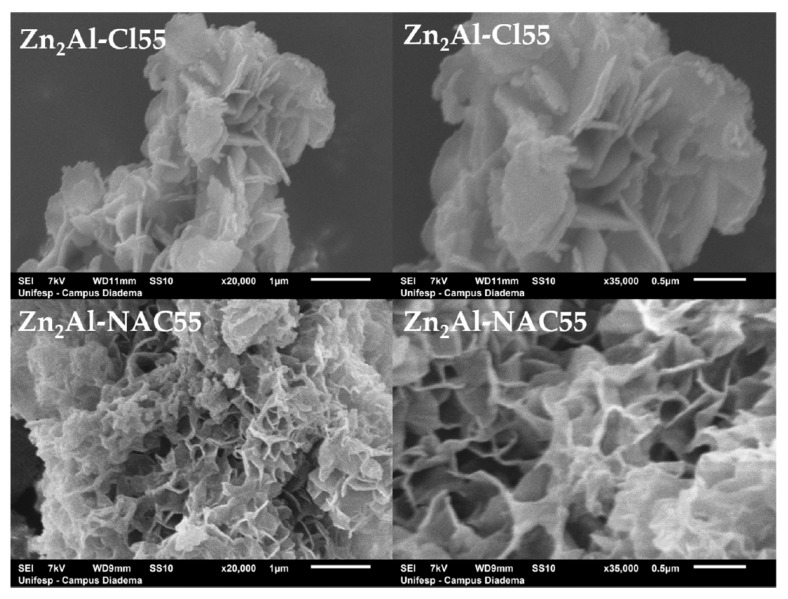
SEM imagens of Zn_2_Al–Cl55 and Zn_2_Al–NAC55 materials in different magnifications: ×20k (**left**) and ×35k (**right**).

SEM images of Zn_2_Al–NAC55 and Zn_2_Al–Cl55 materials in two distinct magnifications showed platelet-shaped particles (Figure 6). The influence of NAC is evident in the aggregation pattern of the platelets with the observation of a more open arrangement (like a foamy sponge) and is associated with flexible particles.

### 3.2. Vibrational Spectroscopic Characterization of Zn_2_Al–NAC and NAC Salt Samples

According to the curves of distribution of NAC chemical species in the pH range 0–14 (Figure S4) [75], at pH values between 5 (pKa_1_ = 3.24) and 8 (pKa_2_ = 9.52), the species with the deprotonated carboxylate group, the mono-anion (NAC)^−^, is predominant. At pH values higher than the pKa_2_, NAC presents both carboxylic and thiol groups deprotonated and the contribution of the dianion (NAC)^2−^ increases strongly. The vibrational spectroscopy should be sensitive to such structural modifications and, for this reason, NAC in NaOH aqueous solutions at four different pH values (7.5, 8.5, 9.5, and 11) were prepared and freeze-dried to record their vibrational spectra. FTIR and Raman spectra of NAC and their associated sodium salts were shown in Figures S5 and S6, respectively, and also the NAC after solubilization in water (without the pH value adjusting) and freeze-drying.

Deprotonation of NAC promoted the disappearing of the band at about 1713 cm^−1^, assigned to the C=O stretching of the carboxylic group, and the emerging of bands related to the carboxylate group at about 1580–1585 cm^−1^ (antisymmetric stretching of -COO^−^) and about 1390–1400 cm^−1^ (symmetric stretching of -COO^−^) (Figure S5). A decrease in the intensity of the band at ca. 2550–2545 cm^−1^, assigned to the S-H stretching, was observed when the pH value was increased. Similarly, the band related to the C-S stretching was shifted from 695 to 685 cm^−1^ (Figure S6).

**Figure 7:**
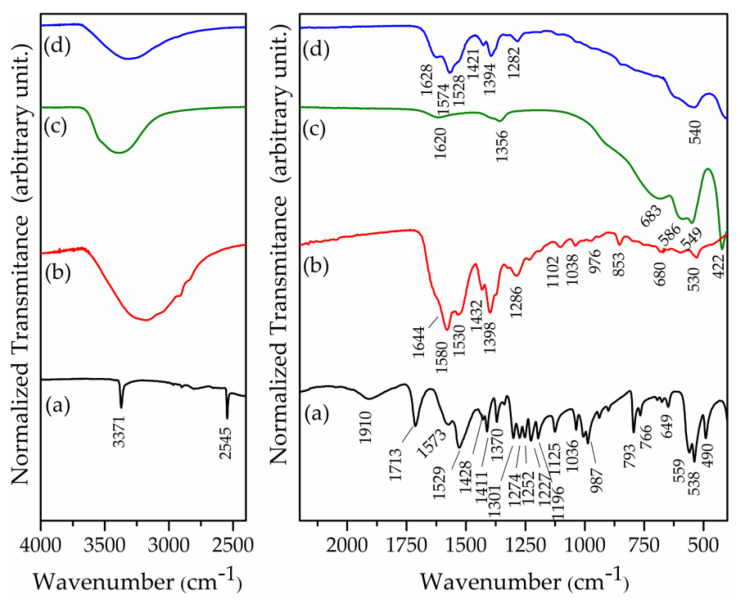
FTIR spectra of (**a**) NAC (polymorph I), (**b**) NAC–pH = 11, (**c**) Zn_2_Al–Cl55, and (**d**) Zn_2_Al–NAC55.

**Figure 8:**
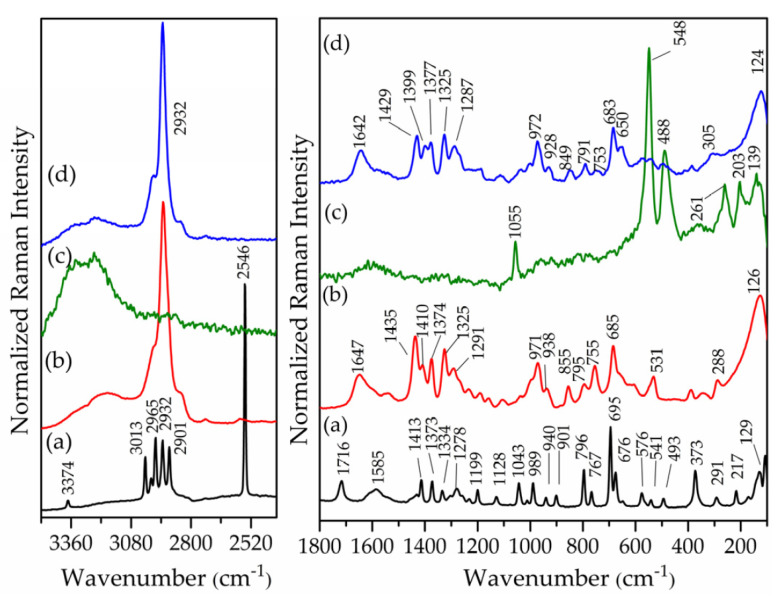
FT–Raman spectra of (**a**) NAC (polymorph I), (**b**) NAC–pH = 11, (**c**) Zn_2_Al–Cl55, and (**d**) Zn_2_Al–NAC55.

The FTIR and FT–Raman spectra of LDH intercalated with NAC and chloride anions are presented in Figure 7 and Figure 8, respectively. For comparison, the spectra of NAC and NAC-pH11 sample are also shown. The FTIR spectrum of Zn_2_Al–Cl55 showed bands in the 3500–3400 cm^−1^ and 1620 cm^−1^ regions attributed to the O-H (from the layers and water) stretching and to the deformation of H_2_O, respectively (Figure 7). In the low-energy region, the Zn_2_Al–Cl55 spectrum presented bands at 422 and 549 cm^−1^ related to Al–O–H and Zn–O–H translations modes, respectively [54]. The Raman spectrum of Zn_2_Al–Cl55 sample (Figure 8) presented bands at 488 and 548 cm^−1^ attributed to Al–O–Al and Zn–O–Al stretching, respectively [54]. The vibrational spectroscopic profiles of Zn_2_Al–NAC55 were rather similar to the spectra of NAC–pH = 11 sample, strongly suggesting that the drug was intercalated in its dianion form (NAC)^2−^ despite the pH of 7.5 applied during the synthesis, probably as a consequence of the highly alkaline character of the interlayer region (see Figure S4).

### 3.3. 13C–NMR Characterization of Zn_2_Al–NAC55 Sample 

**Figure 9:**
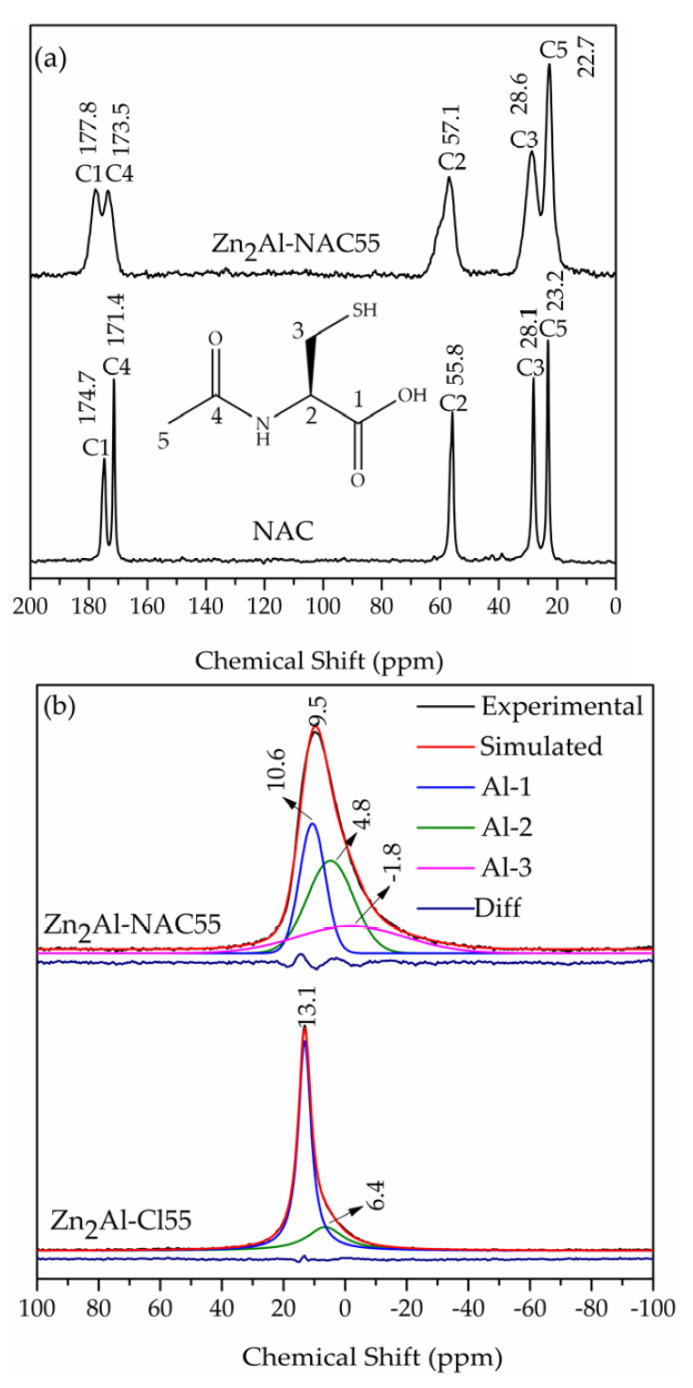
(**a**) ^13^C–CPMAS NMR spectra of NAC (polymorph I) and Zn_2_Al–NAC55 samples and (**b**) ^27^Al–MAS NMR spectra of Zn_2_Al–NAC55 and Zn_2_Al–Cl samples. Deconvolution of peaks of ^27^Al–NMR was performed using the Fityk program version 0.9.8. Diff = difference between experimental and simulated spectrum.

The solid-state ^13^C–NMR spectrum of Zn_2_Al–NAC55 is exhibited in Figure 9a. The chemical integrity of the intercalated species was confirmed by the presence of the five resonance peaks characteristic of the NAC molecule [73]. Chemical shifts for free NAC powder compared to NAC inserted into LDH are as follows: C1 (174.7/177.8), C2 (55.8/57.1), C3 (28.1/28.6), C4 (171.4/173.5), and C5 (23.2/22.7). Most significant shifts are related to C1 and C4 and both peaks shift to a higher frequency when NAC is intercalated with deprotonated carboxylic group C1 (Δδ = 3.1 ppm) [47,76], corroborating FTIR data, while for C4, the shift suggests that NAC amide group is involved in hydrogen bonds when interleaved compared to the free NAC. 

The peaks for C3 and C5 atoms of intercalated NAC were broader, but they did not shift when compared to the free NAC (Figure 9a). Deprotonation of the thiol group could result in a chemical shift of the C3 atom, but it was not observed. A chemical shift (Δδ) of about 3–8 ppm for C3 was noticed when the sulfur atom is coordinated to soft Lewis acid such as Pt(II), Au(I), or Ag(I) [77,78,79]. When NAC is coordinated to Zn^2+^ (an intermediated Lewis acid) by the deprotonated thiol group, the shift Δδ of C3 atom (in D_2_O at pD equal 6.4) compared to NAC in the same conditions was of 2.1 ppm [80]. Hence, the NAC coordination to Zn^2+^ was not supported by NMR data because the C3 peak did not shift after the drug intercalation. No peak was observed at approximately 170 ppm, a region for intercalated carbonate ions [81], indicating that the Zn_2_Al–NAC55 material was not contaminated with carbonate, as also noticed by Raman spectroscopy with the absence of its characteristic stretching band at about 1055 cm^−1^. 

The ^27^Al-NMR spectrum of the Zn_2_Al–NAC55 was recorded to evaluate the Al coordination sphere (Figure 9b). The chemical displacement value of 9.5 ppm indicated the presence of aluminum with six coordination number, Al^(VI)^, because the chemical shift values for octahedral aluminum range from −10 to 15 ppm [82]. The full width at half maximum (FWHM) of the peak was equal to 15.9 ppm and its shape was slightly asymmetrical (a shoulder was observed in the lower frequency part). This peak broadening could be explained by the fact that MAS is not averaging the second order quadrupolar interaction of ^27^Al (I = 7/2) and/or indicating a slight distortion of the metal site. For the latter, the distortion may occur from a strong tethering/bonding with an interleaved species or a disordered cation distribution within the LDH sheets. In the first hypothesis, a strong bonding should correspond to a weakening in the 6-coordination as explained by a grafting process turning a 6-coordinate to a 5 + 1, thus shifting the contribution towards lower-field values. As the opposite shift was observed (from about 13 to 9 ppm if chloride is replaced by NAC), this hypothesis can be discarded.

A perfectly ordered LDH layer comprises 6 surrounding Zn^2+^cations for each Al^3+^ cation, as expressed by [Al(OZn)_6_]. According to the literature [83,84], if a structural disorder occurs in the cation distribution within the LDH layers, some local sites should appear such as [Al(OZn)_5_(OAl)] or [Al(OZn)_4_(OAl)_2_]. The deconvolution of the peak at 9.5 ppm of Zn_2_Al–NAC55 suggested that Al^(VI)^ ions were in distorted sites, most probably as [Al(OM)_5_(OAl)] and [Al(OM)_4_(OAl)_2_)], identified as Al-1 (peak area = 31.5%), Al-2 (peak area = 40.6%), and Al-3 (peak area = 27.9%), respectively, in Figure 9b. The deconvolution of the peak at 13.1 ppm of Zn_2_Al–Cl55 sample suggested the existence of a smaller number of Al sites, identified as Al-1 (peak area = 75.3%) and Al-2 (peak area 24.7%), when compared to the LDH–NAC sample. Therefore, the chloride intercalation promotes a smaller distortion in the metal cation sites than the NAC presence in the interlayer region.

### 3.4. Thermal Analysis Data of Zn_2_Al-NAC55 Sample

For comparison purposes, TGA/DSC and DTG–MS curves of NAC and NAC–pH = 11 samples are shown in Figure S7; the interpretation of the thermal profiles is in the Supporting Materials file. The total weight loss observed after heating NAC–pH = 11 up to 1000 °C is 53.1%, indicating that part of sulfur from the organic drug was lost as SO_2_ at about 250 °C (as observed in the MS curve of NAC in Figure S7) and part was in the calcination residue as sulfate salt (the thermal decomposition of Na_2_SO_4_ is over 1000 °C) [85]. Considering the proposed formula Na_2_(C_5_H_7_NO_3_S)0.45H_2_O for the NAC–pH = 11 salt (exp.: 3.8% H_2_O; calc.: 3.8% H_2_O), a residue consisting of 11.5% of Na_2_O and 39.6% of Na_2_SO_4_ was expected considering the decomposition reaction (Equation (1)):

Na_2_(C_5_H_7_NO_3_S)0.45H_2_O + 8.5O_2_ → 0.6Na_2_SO_4_ + 0.4Na_2_O + 5CO_2_ + 0.4SO_2_ + NO_2_ + 3.95H_2_O (1)

**Figure 10:**
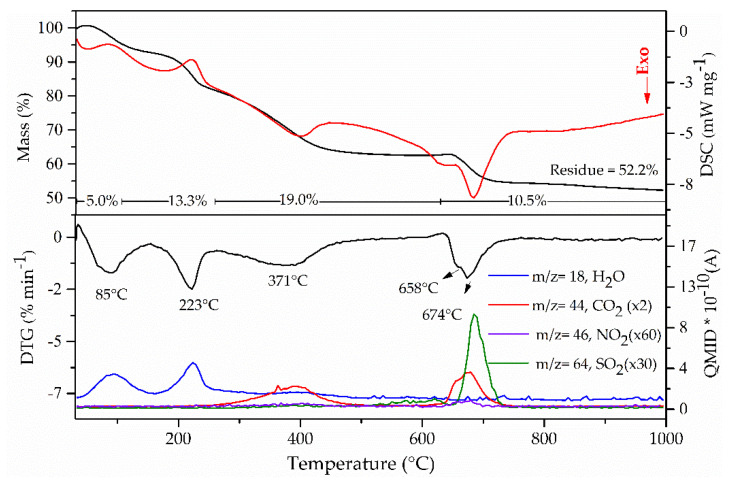
TG/DSC (**upper**) and DTG-MS (**lower**) curves of Zn_2_Al–NAC55 sample.

The thermal composition of the NAC dianion was modified when intercalated into LDH, resulting in four thermal events for the Zn_2_Al-NAC55 sample, as shown in Figure 10. The first event (T_initial_ = 53 °C, DTG peak at 85 °C), an endothermic process, was associated with the sample dehydration, while the second one (T_initial_ = 180 °C, DTG peak at 223 °C) was related to the dehydroxylation of LDH layers with the release of water molecules, as reported for other zinc-based LDH [42,47]. The third event (T_initial_ = 291 °C, DTG peak at 371 °C) was assigned to the beginning of NAC thermal decomposition, evidenced by the loss of CO_2_ molecules. In the fourth step (T_initial_ = 646 °C, DTG peaks at 658 and 674 °C), the release of CO_2_ and SO_2_ molecules was detected, as shown by the MS curves (Figure 10). 

For comparison purposes, the TGA/DSC and DTG–MS curves of Zn_2_Al–Cl55 are shown in Figure S8, while the data discussion was reported in previous work [42]. The residue formed in the Zn_2_Al–NAC55 decomposition was a mixture of ZnO and spinel (ZnAl_2_O_4_) phases, attested by the XRD pattern of the residue. The presence of a non-crystalline sulfate phase in the residue was discarded because its FTIR spectrum did not show bands assigned to this anion, indicating that the sulfur element in the NAC structure was completely converted into a volatile compound. However, the release of SO_2_ at about 700 °C showed that the metal cations from the layers stabilize sulfur species (probably the sulfate ion). Aluminum and zinc sulfates are decomposed at temperatures higher than 500 °C [85]. 

### 3.5. Composition of Zn_2_Al–NAC55 Sample

The chemical elemental analysis (carbon, hydrogen, nitrogen, and metal cations) and the water percentage obtained from TGA for Zn_2_Al–NAC55 were the following: Zn/Al molar ratio = 2.04, 10.20% C, 2.30% N, and 5.0% H_2_O. Considering the carbon amount in the sample, the loading capacity of Zn_2_Al–NAC55 DDS was equal to 27.3% in mass. Considering vibrational spectroscopy and XRD data, NAC was intercalated as the dianion species (i.e., with deprotonated carboxylic and thiol groups). As mentioned in item 3.1, the two samples prepared in this work had an NAC/Al molar ratio equal to 0.65 (Zn_2_Al–1.3NAC55) and 0.68 (Zn_2_Al–NAC55). The excess electric charge of LDH layers is related to the presence of Al^3+^ and it is equal to the number of the trivalent ions. Hence, an NAC/Al molar ratio equal to 0.5 was expected since NAC is present as divalent anion in Zn_2_Al–NAC55 samples. XRD data suggested that Zn_2_Al–NAC55 is a single crystalline phase. Hence, the small excess of NAC can be an amorphous material such as Na_2_NAC adsorbed in the crystalline hybrid material. The proposed composition [Zn_2.04_Al(OH)_6.08_](C_5_H_7_NO_3_S)_0.5_]·1.1H_2_O plus 0.18 Na_2_NAC gives an R value equal to 2.04, 10.16 %C, 2.37 %N, and 4.9 %H_2_O, values very close to those ones obtained experimentally. In this case, the synthesized sample could have 9.3% in mass or 15% in mol of the drug salt of sodium or 7.2% in mass of non-intercalated NAC^2−^.

The presence of the NAC dianion in the sample, as indicated by vibrational spectroscopy, is intricate. At the beginning of the Zn_2_Al–NAC55 synthesis, the amount of NAC was higher than the amount of metal cations and a reaction of complexation should occur. Complexes of Zn:NAC equal to 1:1 and 1:2 proportions can be formed [86]. Zinc is an intermediate Lewis acid and can coordinate with the NAC sulfur atom (*S*-coordination) or form a bidentate ligand (*S,O*-coordination), preferentially in 4-fold coordination. The speciation diagram for Zn-NAC complexes obtained by the pH−potentiometric technique indicated that the major species at pH 7.5 is [Zn(NAC)_2_]^2−^ (log*β* is about 12) [86]. As the synthetic reaction for LDH formation progresses, metal hydrolysis reactions also occur; [Zn(NAC)_x_(OH)_n_]^2−2x−n^ species can be formed, involving two kinds of ligands coordinated to a four-fold zinc cation. Therefore, several metal complex species should co-exist in solution at pH around 7.5. As the time of synthesis advances, the precipitation of Zn_2_Al–NAC55 material resulted from olation reactions among octahedral hydroxide complexes of Zn^2+^ and Al^3+^; the positive charge of layers is neutralized by NAC dianions. Despite the pH value of the medium, the stability of the intercalated drug could be enhanced by hydrogen bonding among the confined (NAC)^2−^ ions. 

### 3.6. Characterization of Mg_2_Al–NAC Sample

Synthetic parameters were varied aiming for the NAC intercalation into LDH of magnesium and aluminum composition. XRD patterns recorded for materials prepared as described in the Experimental section and isolated using an NAC/Al^3+^ molar ratio equal to 0.5 or 2, applying or not a post-synthesis thermal treatment for 24 h at 90 °C, produced materials with d(003) basal spacing around 7.53 Å (Figure S9). These data indicated that NAC was not intercalated into LDH. The FTIR spectrum of Mg_2_Al–NAC sample showed the bands related to the carboxylate group at 1574 cm^−1^ and 1370 cm^−1^, denoting the presence of NAC in the sample (Figure S10). The main remark about the spectral profile of Mg_2_Al–NAC was the identification of a Raman band at 508 cm^−1^ (Figure S10), assigned to the S-S stretching mode [57]. This result implied that NAC was oxidized in the Mg_2_Al–NAC sample. At first glance, this fact could be associated with the pH value of the LDH synthesis, but neither NAC–pH = 9.5 nor NAC–pH = 11 samples presented the band at about 508 cm^−1^. The oxidation of the thiol group of an organic compound (mercaptosuccinate) during LDH synthesis has already been reported [87,88]. The solid-state ^13^C–CPMAS NMR spectrum of Mg_2_Al–NAC showed the expected five peaks and other ones. C2 and C3 peaks assigned to thiol group oxidation were observed at 53.9 and 36.9 ppm, respectively (Figure S11).

### 3.7. In Vitro NAC Release Kinectics Experiments

**Figure 11:**
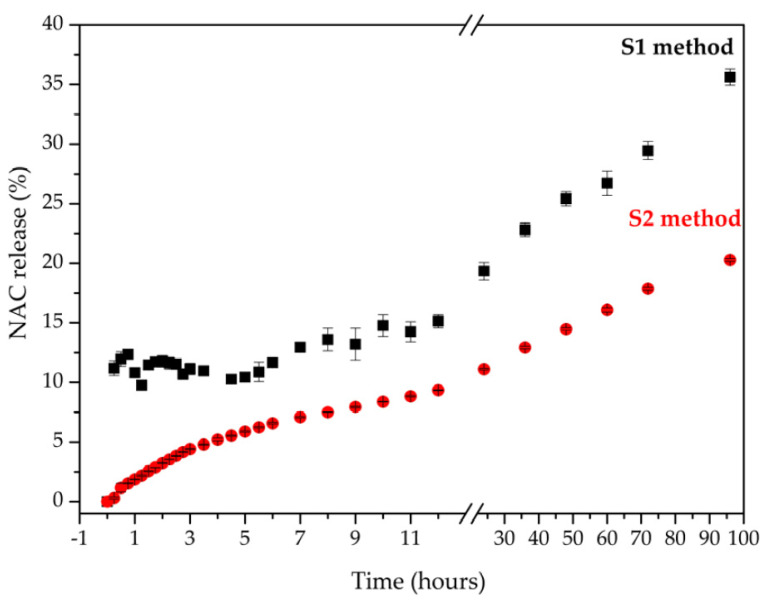
Release data of NAC with respect to time from Zn_2_Al–NAC55 using S1 (Apparatus 1—basket, 50 rpm) and S2 (without agitation) methods (n = 3).

The cumulative amounts of NAC released from Zn_2_Al–NAC55 versus time using the method with agitation (S1 method) and without agitation (S2 method) in SBF medium at 37 °C are shown in Figure 11. After 96 h, 35.6 ± 0.7% of NAC was released under agitation (S1 method), while 20.3 ± 0.1% was observed without agitation (S2 method). The ions tend to migrate from a region of high concentration (simulated physiological solution) to a region of low concentration (the tablet environment and the interlayer region). The diffusion from a site of high to a low concentration, as occurs at the beginning of the release experiment, is more pronounced than the opposite, which occurs mainly in situations of static conditions. However, the solution agitation nearby the tablet can enhance the ions flux with time, which was observed in this work (Figure 11). Considering that NAC is readily soluble in water in its neutral form (solubility equal to 100 mg mL^−1^) [28], the obtained data suggest a modified release profile by intercalation process in a layered structure. The S1 method agitation process favors sink conditions and allows a higher release when compared to S2 method. Mathematical models were applied to the release data to shed light on the delivery mechanism and evaluate the role of the medium agitation. 

The concentrations of cations in the solutions after the end of the experiment were quantified to verify if there was leaching of the metal cations from the DDS material. After 96 h of testing applying S1 and S2 methods, the concentrations of zinc cations were 0.45 mg L^−1^ (0.17% of the metal in the tablet) and 1.57 mg L^−1^ (0.025%), respectively. In both conditions, the aluminum cation was not detected because its amount was lower than the limit detection (LD < 0.01 mg L^−1^). Therefore, the leaching of the metal cations of the layers was minimal, indicating that the drug release mechanism was not related to the carrier solubilization in the SBF medium. The release process studied in this work can be classified as ion exchange, similar to the DDS materials based on ion exchange resins, in which the drug is delivered by the replacement of physiological ions in the medium [89].

The application of mathematical models to fit experimental release data allows understanding the mechanisms involved in the release of a particular drug from a formulation, as well as obtaining parameters about the process rate and maximum amount of drug released, among other information. The kinetics of drug release from drug delivery systems can follow different behaviors, such as the first-order model, which corresponds to a high dose release in a quick and immediate form or a slow, constant and controlled release behavior, defined by zero-order, Higuchi, Hixson–Crowell, and other models [62,63,64,65,89]. The graphics related to the mathematical models acceptable to DDS studied in this work are shown in Figure S12.

The release of the drug can occur in a controlled way by different processes, such as diffusion through an inert carrier, diffusion through a membrane or a hydrophilic gel, osmosis, and ion exchange [89,90]. According to the results shown in Table 2, the dependent models that best define the NAC release process from the drug delivery system developed in this work were Hixson–Crowell (R^2^ = 0.9786) when using the S1 method (basket, 50 rpm) and Higuchi (R^2^ = 0.9766) with S2 (without agitation). Both the Hixson–Crowell and Higuchi models are applied to controlled release systems. However, in the Hixson–Crowell model, the drug release rate is limited by drug dissolution and not by diffusion. Considering the experimental data about the insignificant amount of zinc and aluminum ions leached from the tablets after 96 h, the release of NAC occurs by anion exchange, as discussed above. The release phenomena should be different when using S1 or S2 methods because agitation favors sink conditions. 

The Hixson–Crowell and Higuchi equations kinetic models were used to determine T50 (time to release 50% of the drug in the DDS) and T90 (time to release 90% of the drug in the DDS) values for the S1 and S2 methods, respectively. T50 is about 154 and 565 h for the S1 and S2 methods, respectively, while T90 is approximately 453 and 1854 h. According to the values of T50 and T90, the DDS allows a prolonged release, with even NAC showing high solubility. Then, the kinetics data indicated a controlled, constant, and slow-release behavior of the drug from LDH–based DDS that can provide dose maintenance, allowing the achievement of the desired drug concentration in a target tissue. 

**Figure 12:**
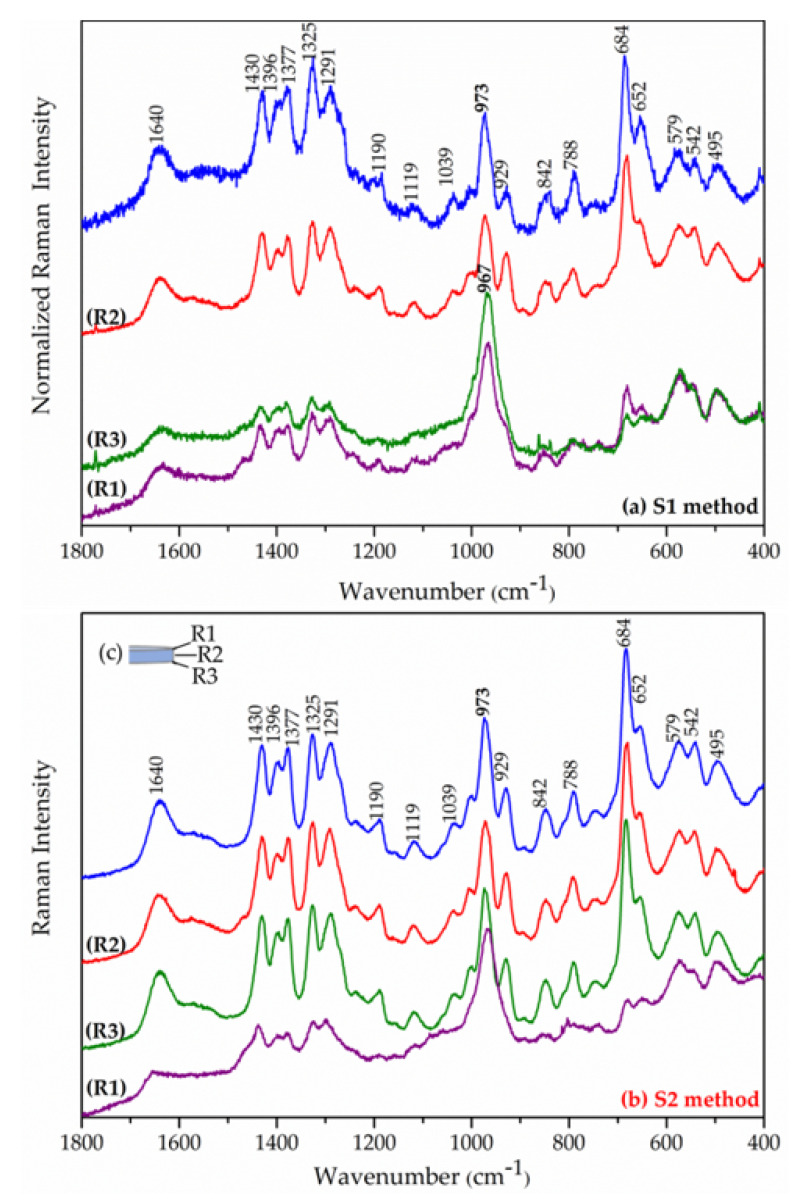
Raman spectra along the cross section of Zn_2_Al–NAC55 tablets after 96 h of release assay: (**a**) with agitation (S1 method), (**b**) without agitation (S2 method), and (**c**) regions of the tablet where the spectra were collected from the upper surface to the inferior part: R1 and R3 correspond to the external regions of the tablet, while R2 corresponds to the internal region. The spectrum of original Zn_2_Al–NAC55 powder is shown in the blue line.

Figure 12 shows the Raman spectra of the stratigraphic analysis obtained from the points (R1) to (R3) of the tablets after 96 h. To verify the changes in the internal regions of the tablet after the release experiment, spectra were obtained in regions of its meridional section. In the external region, the spectra of the tablets showed an intensification of the band around 973 cm^−1^ in relation to the other ones. This band is assigned to the ν_s_(P-O) mode of the HPO_4_^2−^ anion [91,92]. After 96 h, the internal region of the tablets was unmodified, suggesting that the NAC anion exchange by hydrogen phosphate did not reach the interior region of the tablet yet, as indicated by the release profile (Figure 11). The SBF solution has other anions that can promote the NAC release by ion exchange such as chloride which is in higher concentration in the simulated physiological medium than HPO_4_^2−^ anion, which cannot be identified by Raman spectroscopy. 

**Figure 13:**
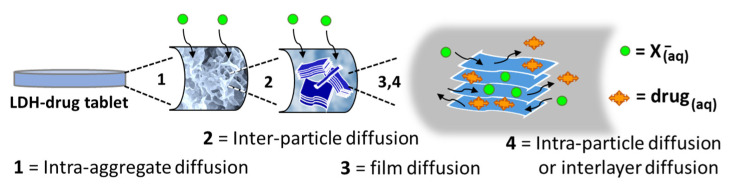
Schematic representation of NAC release from DDS based on Zn_2_Al–NAC55.

Figure 13 presents a simple schematic representation of the NAC release process from LDH: initially, the SBF solution moistens the LDH carrier system promoting an intra-aggregate ion diffusion; next, ion diffusion reaches an inter-particle region; finally, ions from the SBF solution migrate into a diffusion film coating the primary particles and ion exchange of NAC by anions such as HPO_4_^2−^ takes place by intra-particle or interlayer diffusion.

## 4. Conclusions

LDH–NAC hybrid material composed of Zn/Al cations was successfully synthesized by co-precipitation method and an improved crystallinity was observed when the temperature of synthesis was raised to 55 °C. XRD data confirmed the formation of a crystalline layered material with an interlayer distance of 16.38 Å, resulting from a confined/constrained NAC bilayer arrangement, indicated by the calculated Patterson electrons density map. FTIR, Raman, and ^13^C–NMR spectroscopic data confirmed the integrity of NAC after intercalation, and vibrational spectroscopy also showed that NAC had two negative charges when confined into LDH. On the other hand, the composition Mg/Al did not stabilize the drug but promoted its partial oxidation. The ^27^Al-NMR spectrum of Zn_2_Al–NAC55 showed peaks associated with distinct aluminum sites with a coordination number of six but did not indicate the coordination of NAC to the hydroxylated layers. These results, together with chemical and thermal analysis, allowed the proposition of the chemical formula of the Zn_2_Al–NAC55 DDS, whose loading capacity was equal to 27.3% in mass. The in vitro release of NAC from the Zn_2_Al–LDH carrier with and without agitation was 35.6 ± 0.7% and 20.3 ± 0.1%, respectively, after 96 h in SBF medium. The release profile fits the Hixson–Crowell and Higuchi kinetic models for the S1 and S2 methods, respectively. Kinetics data indicated that NAC is released from the Zn_2_Al LDH in a controlled, mostly constant, and slow mode, contrasting with the free drug, which has high solubility in water. Raman spectra along the cross section of the Zn_2_Al–NAC tablets recorded after the release assays allowed the elucidation of the release process, driven by ion diffusion and preservation of LDH structural integrity The LDH carrier containing the antioxidant NAC may be of interest in the pharmaceutical and medical fields as an implantable DDS material for tissue engineering because of its controlled and slow-release properties highlighted in this work, as well as the biological activity of NAC and zinc ions in tissue repair. 

## Figures and Tables

**Table 1:** Mathematical models of drug release used in this work.

Model	Mathematical Equation
Zero order	$1-\frac{M_{t}}{M_{0}}={M_{0}-k}_{0}t$
First order	$\ln\left(M_{0}-M_{t}\right)={\ln M}_{0}-k_{1}t$
Higuchi	$M_{t}=k_{H}\sqrt{t}$
Hixson–Crowell	$\sqrt[{3}]{\left(1-\frac{M_{t}}{M_{0}}\right)}=1-k_{\beta }t$
Baskar	$-\ln\left(1-\frac{M_{t}}{M_{0}}\right)=ln\left(\frac{M_{0}}{M_{0}-M_{t}}\right)=1.59{\left(\frac{6}{d_{p}}\right)}^{1.3}{\left(Dt\right)}^{0.65}$

$M_{t}$ is the amount of NAC released at time *t*; $M_{0}$ is the initial amount of NAC; *k* is the kinetic release constant; *D* is the diffusion constant; $d_{p}$ is particle diameter.

**Table 2:** NAC release kinetics data analysis according to different mathematical models for the full-range time of the experiments.

	S1 Method		S2 Method	
	*R* ^2^	Equation	*R^2^*	Equation
Zero-order	0.8815 *	y = −0.0028x + 0.8967	0.8648 *	y = −0.002x + 0.9601
First-order	0.9112 *	y = 0.014x − 2.1837	0.4522 *	y = 0.0253x − 3.3505
Higuchi	0.941 *	y = 2.5917x + 7.0461	0.9766 *	y = 2.0737x + 0.7088
Hixson–Crowell	0.9786 *	y = −0.0011x + 0.9629	0.8786 *	y = −0.0007x + 0.9866
Baskar	0.9651 *	y = 0.0164x + 0.0875	0.9595 *	y = 0.0112x + 0.0214

*R*^2^ = Correlation coefficient; * *p*-value < 0.002 according to analysis of variance.

## Data Availability

Raw data were generated at University of São Paulo (Brazil) and Université Clermont Auvergne (France). Derived data supporting the results of this study are available from the corresponding author (V.R.L.C.) on request.

## Supplementary Materials

The following supporting information can be downloaded at: https://www.mdpi.com/article/10.3390/pharmaceutics15030955/s1, Figure S1: XRD patterns of LDH–NAC synthesized at distinct experimental conditions: (a) Zn_2_Al–NAC (room temperature and NAC/Al molar ratio = 1), (b) Zn_2_Al–NAC55 (55 °C and NAC/Al molar ratio = 1), and (c) Zn_2_Al–1.3NAC55 (55 °C and NAC/Al molar ratio = 1.3); Figure S2: Comparison between XRD patterns of (a) NAC and its polymorphs, (b) I, and (c) II; Figure S3: Crystalline packaging of polymorphs I and II, obtained in the Vesta version 3 program, from the crystallographic data deposited at Cambridge Crystallographic Data Centre; Figure S4: Curves of distribution of NAC species, where α_i_ is the species fraction, obtained through the *CurTiPOt* program; Figure S5: FTIR spectra of (a) NAC, (b) NAC dissolved in water and posteriorly freeze-dried, (c) NAC–pH = 7.5, (d) NAC–pH = 8.5, (e) NAC–pH = 9.5, and (f) NAC–pH = 11; Figure S6: FT–Raman spectra of (a) NAC, (b) NAC dissolved in water and posteriorly freeze-dried, (c) NAC–pH = 7.5, (d) NAC–pH = 8.5, (e) NAC–pH = 9.5, and (f) NAC–pH = 11; Figure S7: TG–DSC and DTG–MS curves of NAC polymorph I and NAC-pH = 11 samples; Figure S8: TG-DSC and DTG-MS curves of Zn_2_Al–Cl55 sample under atmospheric air; Figure S9: XRD patterns of (a) Mg_2_Al–Cl, (b) Mg_2_Al–NAC, (c) Mg_2_Al–0.5NAC (NAC/Al^3+^ molar ratio used in the synthesis = 0.5), (d) Mg_2_Al–2NAC (NAC/Al^3+^ molar ratio used in the synthesis = 2), (e) Mg_2_Al–NAC_90_24h (aging process at 90 °C for 24 h), and (f) Mg_2_Al–NAC55; Figure S10: FTIR and FT–Raman spectra of (a) NAC, (b) NAC–pH = 9.5, (c) Mg_2_Al–Cl, and (d) Mg_2_Al–NAC; Figure S11. ^13^C–CPMAS NMR spectra of NAC and Mg_2_Al–NAC samples; Figure S12. Kinetic models applied to the Zn_2_Al–NAC55 sample. Table S1: IR and Raman band positions (in cm^−1^) of NAC (polymorph I), NAC–pH = 11.0, Zn_2_Al–NAC55, and their tentative assignment. References [42,73,83] are cited in supplementary materials.
