# Supplementary material for: Development of Dipeptide N–acetyl–L–cysteine Loaded Nanostructured Carriers Based on Inorganic Layered Hydroxides
Source: Pharmaceutics. 2023 Mar 15;15(3):955. doi: 10.3390/pharmaceutics15030955 (PMC10054814; doi:10.3390/pharmaceutics15030955)
Supplement: Supplementary file 1 [file pharmaceutics-15-00955-s001.zip › pharmaceutics-2205301-supplementary.pdf]

**Supplementary Material**

# Development of dipeptide *N*-acetyl-L-cysteine loaded nanostructured carriers based on inorganic layered hydroxides

**Denise Eulálio,[1] Mariana Pires Figueiredo,[1] Christine Taviot-Gueho,[2,3] Fabrice Leroux,[2,3] Cristina Helena dos Reis Serra,[4] Dalva Lúcia Araújo de Faria[1] and Vera Regina Leopoldo Constantino[1*]**

[1] Departamento de Química Fundamental, Instituto de Química, Universidade de São Paulo - USP, Av. Prof. Lineu Prestes 748, CEP 05508-000, São Paulo, SP, Brazil.

[2] Université Clermont Auvergne, Institut de Chimie de Clermont-Ferrand, BP 10448, F-63000 Clermont-Ferrand, France.

[3] CNRS, UMR 6296, ICCF, F-63178 Aubiere, France.

[4] Departamento de Farmácia, Faculdade de Ciências Farmacêuticas, Universidade de São Paulo - USP, Av. Prof. Lineu Prestes 580, CEP 05508-000, São Paulo, SP, Brazil.

* Correspondence: vrlconst@iq.usp.br (V.R.L.C.); Tel.:+55 (11) 3091-9152

## Content

**1.** XRD patterns of LDH-NAC samples and NAC polymorphs
**2.** Crystal structure of NAC polymorphs obtained by XRD
**3.** Speciation curves for NAC species relative to pH value
**4.** FTIR and FT-Raman spectra of NAC samples obtained by freeze-drying of solutions in different pH values
**5.** Thermal analysis data of NAC and NAC-pH=11 samples
**6.** Thermal analysis data of $Zn_2Al$-Cl55 sample
**7.** XRD patterns of $Mg_2Al$-NAC samples obtained at different synthetic parameters
**8.** FTIR and Raman spectra of $Mg_2Al$-NAC and $Mg_2Al$-Cl samples
**9.** $^{13}C$-CPMAS NMR spectra of NAC and $Mg_2Al$-NAC sample
**10.** Kinetic models applied to the of NAC from $Zn_2Al$-NAC55 sample

## 1. XRD patterns of LDH-NAC samples and NAC polymorphs

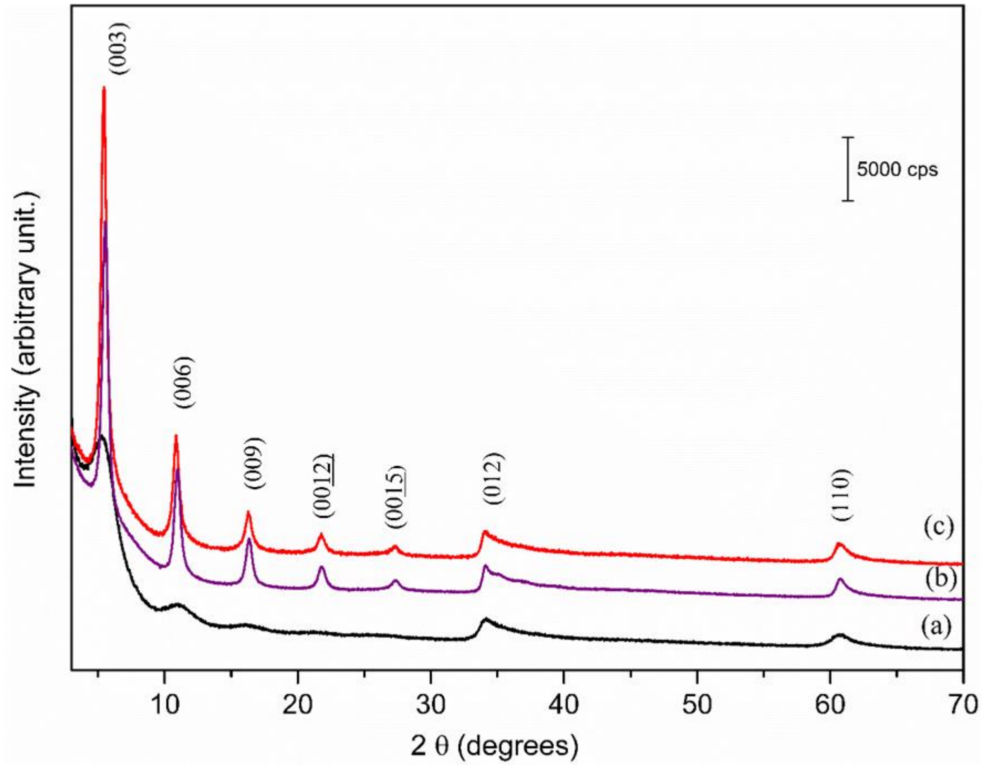


**Figure S1.** XRD patterns of LDH-NAC synthesized under distinct experimental conditions: (a) $Zn_2Al$-NAC (room temperature and NAC/Al molar ratio =1), (b) $Zn_2Al$-NAC55 (55 °C and NAC/Al molar ratio =1), and (c) $Zn_2Al$-1.3NAC55 (55 °C and NAC/Al molar ratio =1.3).

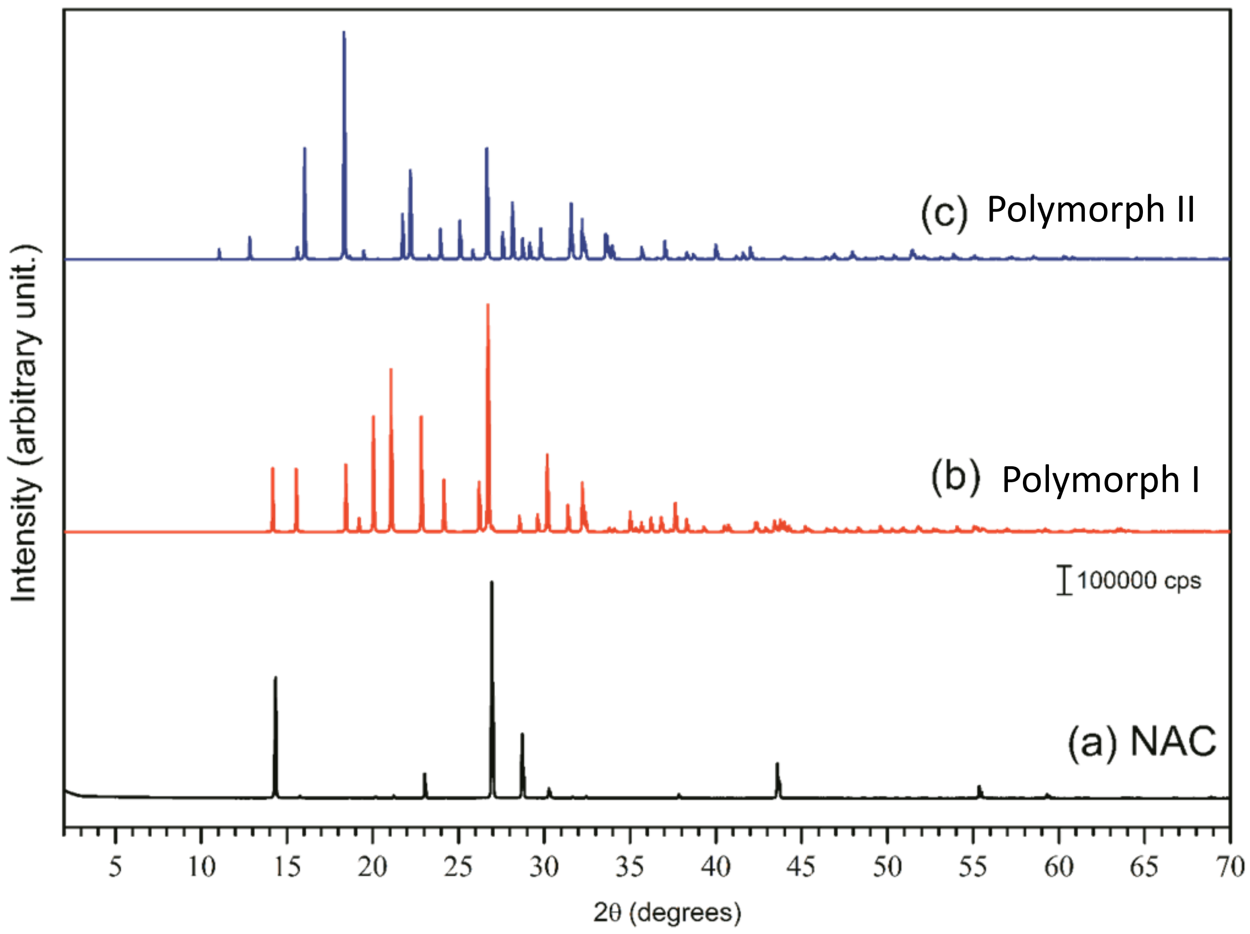


**Figure S2.** Comparison between XRD patterns of (a) NAC used in this work, (b) Polymorph I and (c) Polymorph II.

## 2. Crystal structure of NAC polymorphs obtained by XRD

Polymorph I structure comprises intermolecular interactions between NH•••S (2.82 Å), OCOH•••O=C (1.65 Å), and CH•••OCOH (2.72 Å), and intramolecular interaction between NH•••OCOH (2.26 Å).[1] Polymorph II shows intermolecular interactions of NH•••S (2.97 Å), OCOH•••O=C (1.66 Å), and CH•••OCOH (2.36 Å) and SH•••OCOH (2.15 Å). NAC used in this work is the polymorph I.

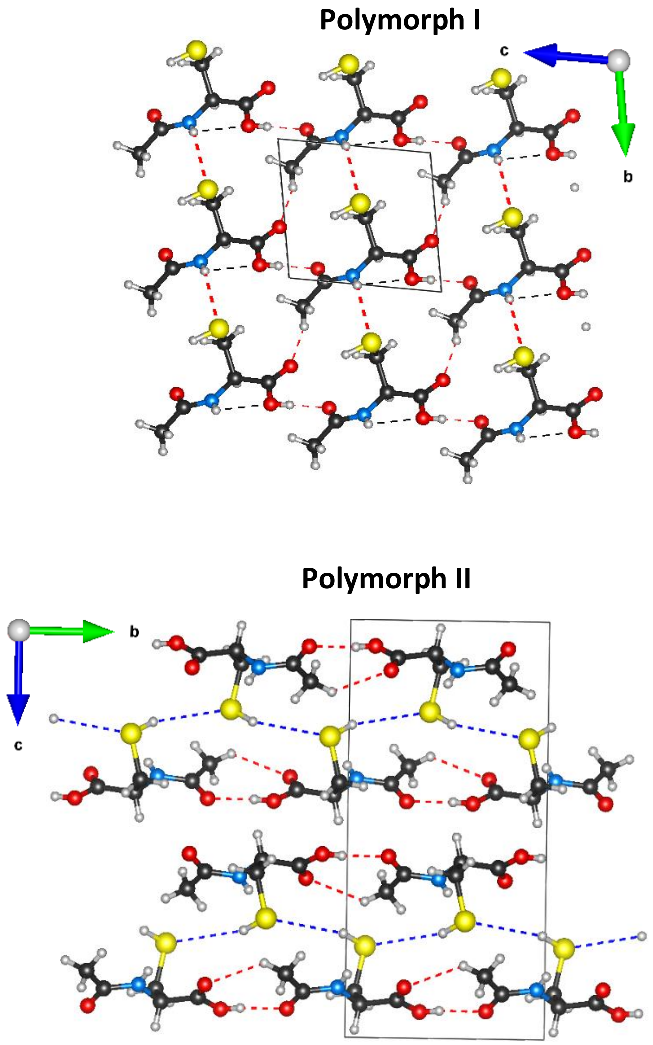


**Figure S3.** Crystalline packaging of polymorphs I and II, obtained in the Vesta version 3 program, from the crystallographic data deposited at Cambridge Crystallographic Data Centre by Kumar e Nagia [1].

## 3. Speciation curves for NAC species relative to pH value

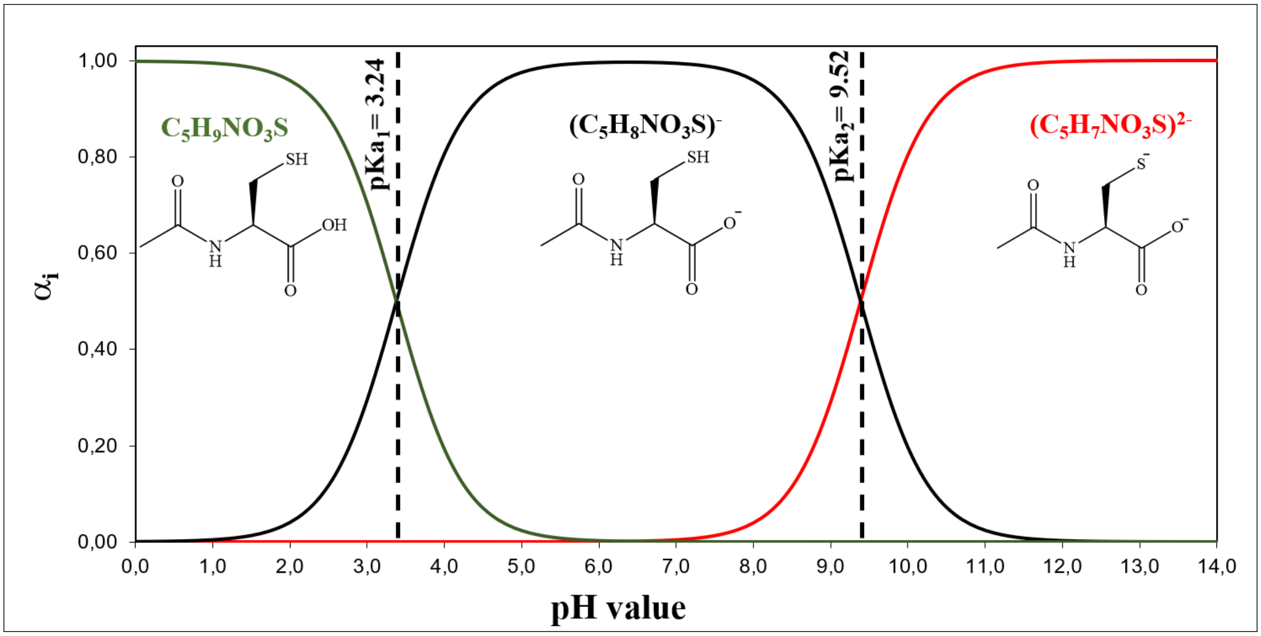


**Figure S4.** Curves of distribution of NAC species, where $\alpha_i$ is the species fraction, obtained through the *CurTiPOt* program, developed by Prof. Dr. Ivano G. R. Gutz, from Instituto de Química - USP.

## 4. FTIR and FT-Raman spectra of NAC samples obtained by freeze-drying of solutions in different pH values

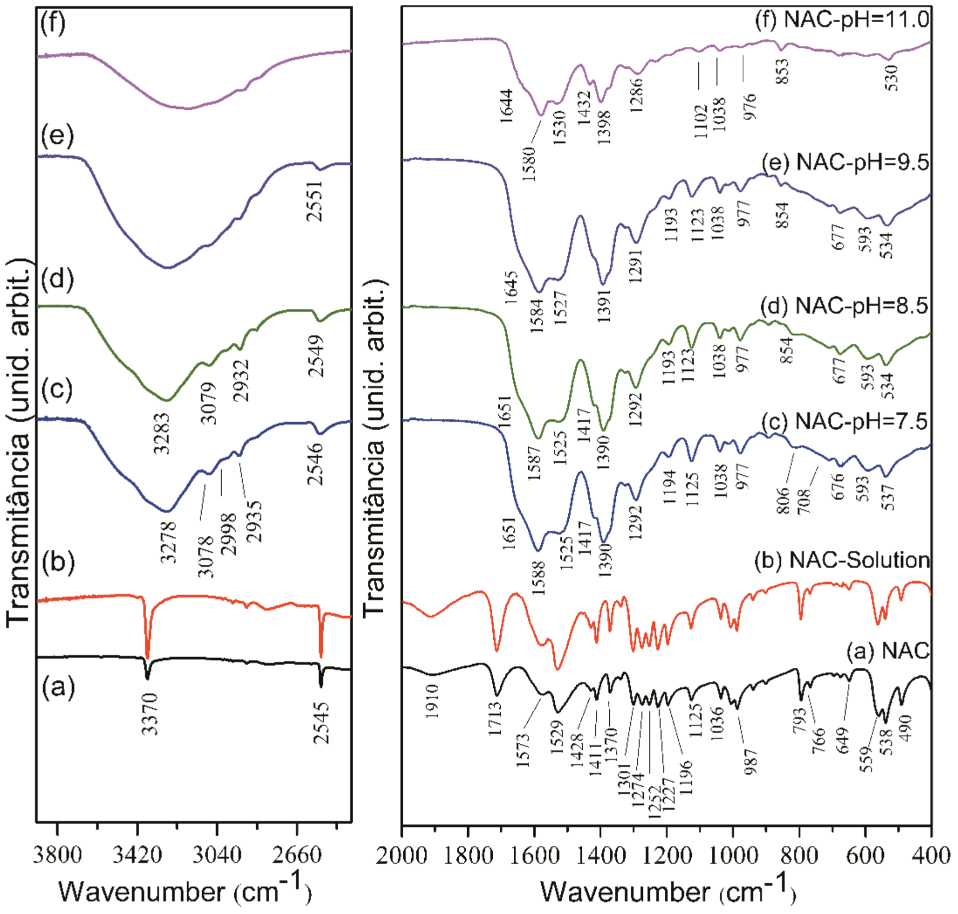


**Figure S5.** FTIR spectra of (a) NAC, (b) NAC dissolved in water and posteriorly freeze-dried, (c) NAC-pH=7.5, (d) NAC-pH=8.5, (e) NAC-pH=9.5, and (f) NAC-pH=11.

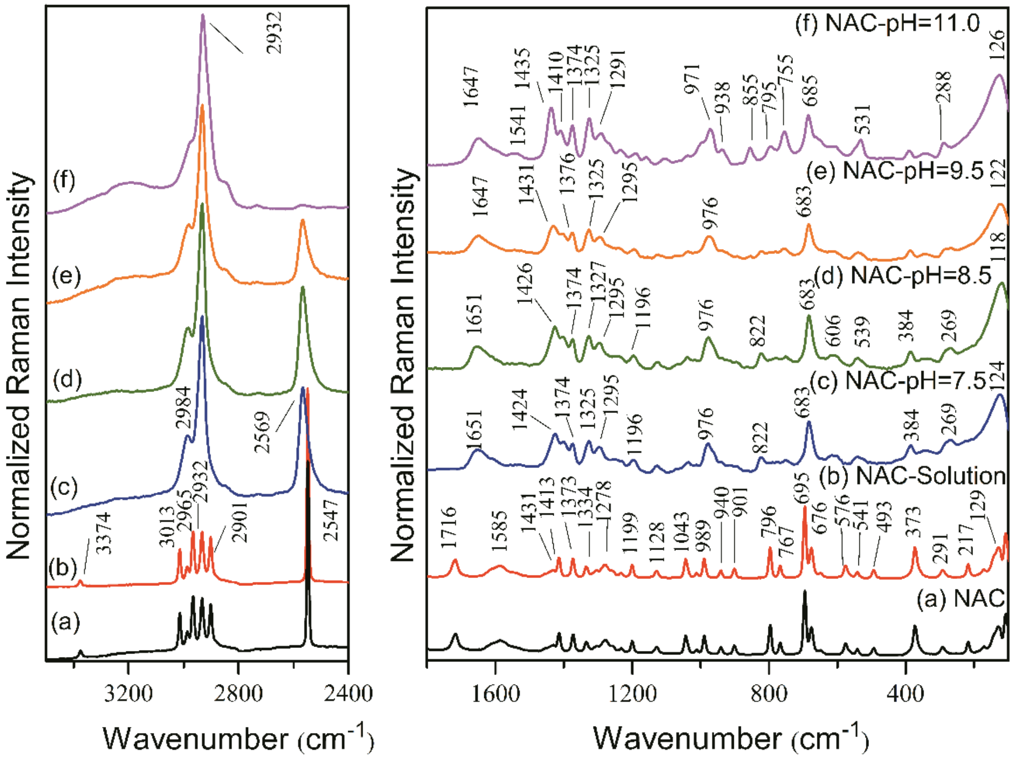


**Figure S6.** FT-Raman spectra of (a) NAC, (b) NAC dissolved in water and posteriorly freeze-dried, (c) NAC-pH=7.5, (d) NAC-pH=8.5, € NAC-pH=9.5, and (f) NAC-pH=11.

**Table S1.** IR and Raman band positions (in $cm^{-1}$) of NAC (polymorph I), NAC-pH=11.0, $Zn_2Al$-NAC55 and their tentative assignment.

| NAC | | NAC-pH=11 | | $Zn_2Al$-NAC55 | | Tentative assignment |
|---|---|---|---|---|---|---|
| FTIR | Raman | FTIR | Raman | FTIR | Raman | |
| 3370 | 3374 | | | | | ν N-H |
| | | | | 3322 | | ν O-H |
| | 3013 | | | | | $\nu_{as}$ C-H ($CH_3$) |
| | 2965 | | | | | $\nu_{as}$ C-H ($CH_2$) |
| | 2932 | 2932 | 2932 | | | $\nu_s$ C-H ($CH_3$) |
| | 2901 | | | | 2932 | $\nu_s$ C-H ($CH_2$) |
| 2545 | 2547 | | | | | ν S-H |
| 1713 | 1716 | | | | | ν C=O (COOH) |
| | | | | 1628 | | δ $H_2O$ |
| 1573 | 1585(b) | 1644 | 1647 | | | ν C=O (amide) |
| | | 1580 | | 1574 | | $\nu_{as}$ $COO^-$ |
| 1529 | | 1525 | | 1528 | | δ N-H |
| | | | | | 1429 | δ $CH_2$ |
| | | 1398 | | 1394 | 1399 | $\nu_s$ $COO^-$ |
| 1274 | 1278 | 1286 | 1291 | 1292 | 1287 | ν C-N |
| | 695 | 680(w) | 685 | | 683 | ν C-S |
| | | | | | 124 | δ O-Zn-O |

a) ν = stretching, δ = bending, s = symmetric, as = antisymmetric
b) (b) = broad; (w) = weak

## 5. Thermal analysis data of NAC and NAC-pH=11 samples

NAC melts at 120°C (endothermic peak) and decomposes in two main events (**Figure S7**). XRD pattern of NAC used in this work is assigned to the polymorph I. The first event has a $T_{initial}$ value equal to 130 °C (DTG peaks at 198 and 216 °C) and the second equal to 440 °C (DTG peak at 559 °C); in both events occur the loss of $H_2O$, $CO_2$, and $SO_2$ molecules. No residue is noticed after the sample heated up to 1000 °C.

The thermal analysis data of NAC-pH=11 sample shows the loss of hydration water molecules from room temperature up to about 200 °C, followed by the decomposition step ($T_{initial}$= 220 °C, DTG peak at 252 °C) (**Figure S7**). The release of $CO_2$ above 700 °C indicates the formation of an intermediate carbon species.

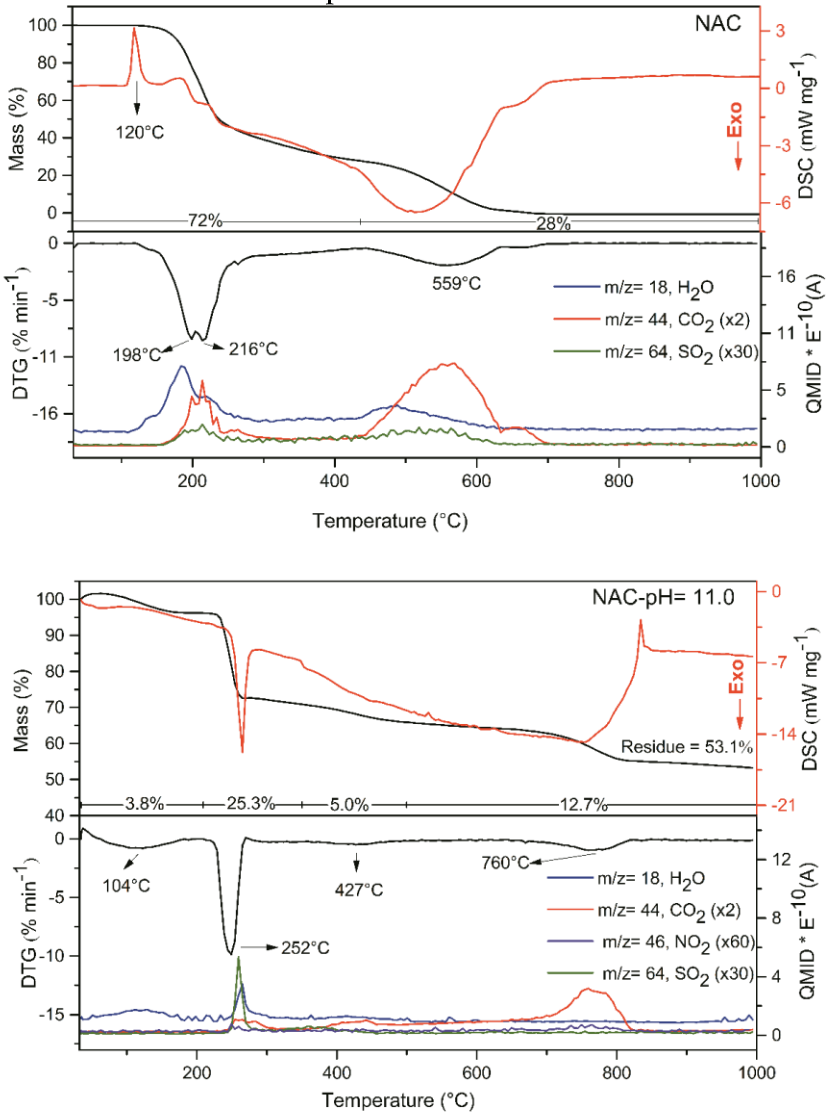


**Figure S7.** TG/DSC and DTG-MS curves of NAC polymorph I (top) and NAC-pH=11 samples (down).

## 6. Thermal analysis data of $Zn_2Al$-Cl55 sample

The assignment of the events occurring in the thermal decomposition of $Zn_2Al$-Cl (shown in **Figure S8**) was previously reported [2]. The amount of HCl released from dehydrochlorination is low and could not be identified in the experimental conditions applied in this work.

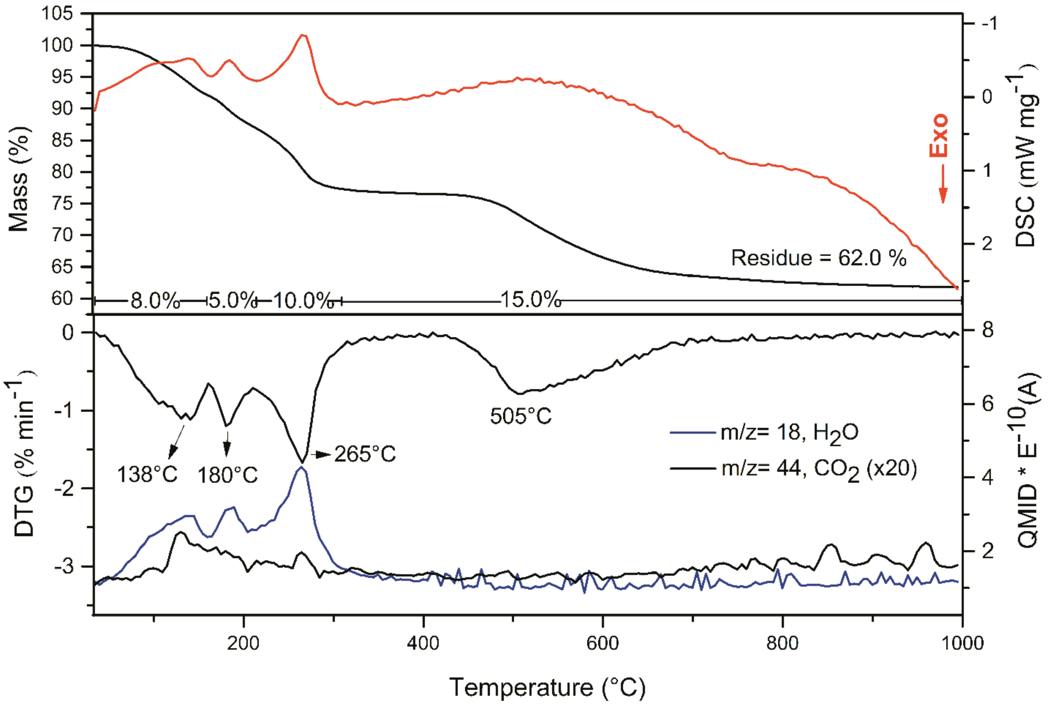


**Figure S8.** TG/DSC and DTG-MS curves of $Zn_2Al$-Cl55 sample under atmospheric air.

## 7. XRD patterns of $Mg_2Al$-NAC samples obtained at different synthetic parameters

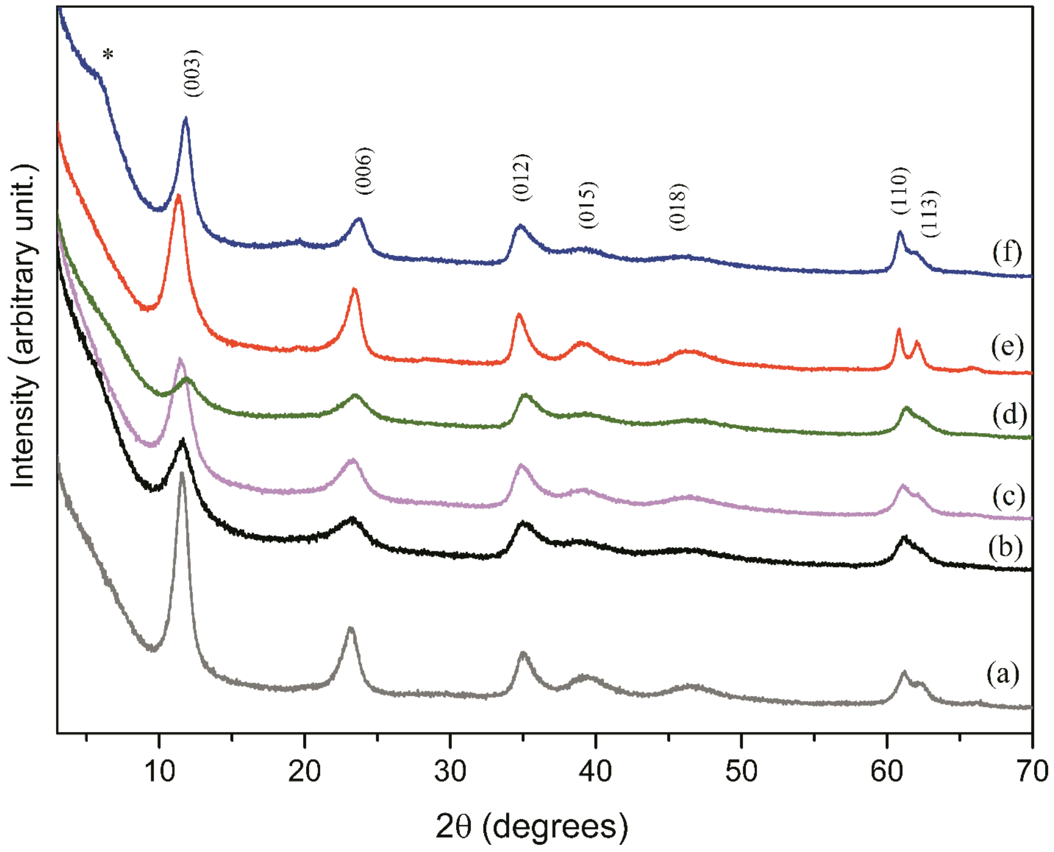


**Figure S9.** XRD patterns of (a) $Mg_2Al$-Cl (b) $Mg_2Al$-NAC (synthesis described in the Experimental section), (c) $Mg_2Al$-0.5NAC (NAC/$Al^{3+}$ molar ratio used in the synthesis = 0.5), (d) $Mg_2Al$-2NAC (NAC/$Al^{3+}$ molar ratio used in the synthesis = 2) (e) $Mg_2Al$-NAC_90_24h (aging process at 90°C for 24 h) and (f) $Mg_2Al$-NAC55 (synthesis described in Experimental section). (*) equipment artefact.

## 8. FTIR and Raman spectra of $Mg_2Al$-NAC and $Mg_2Al$-Cl samples

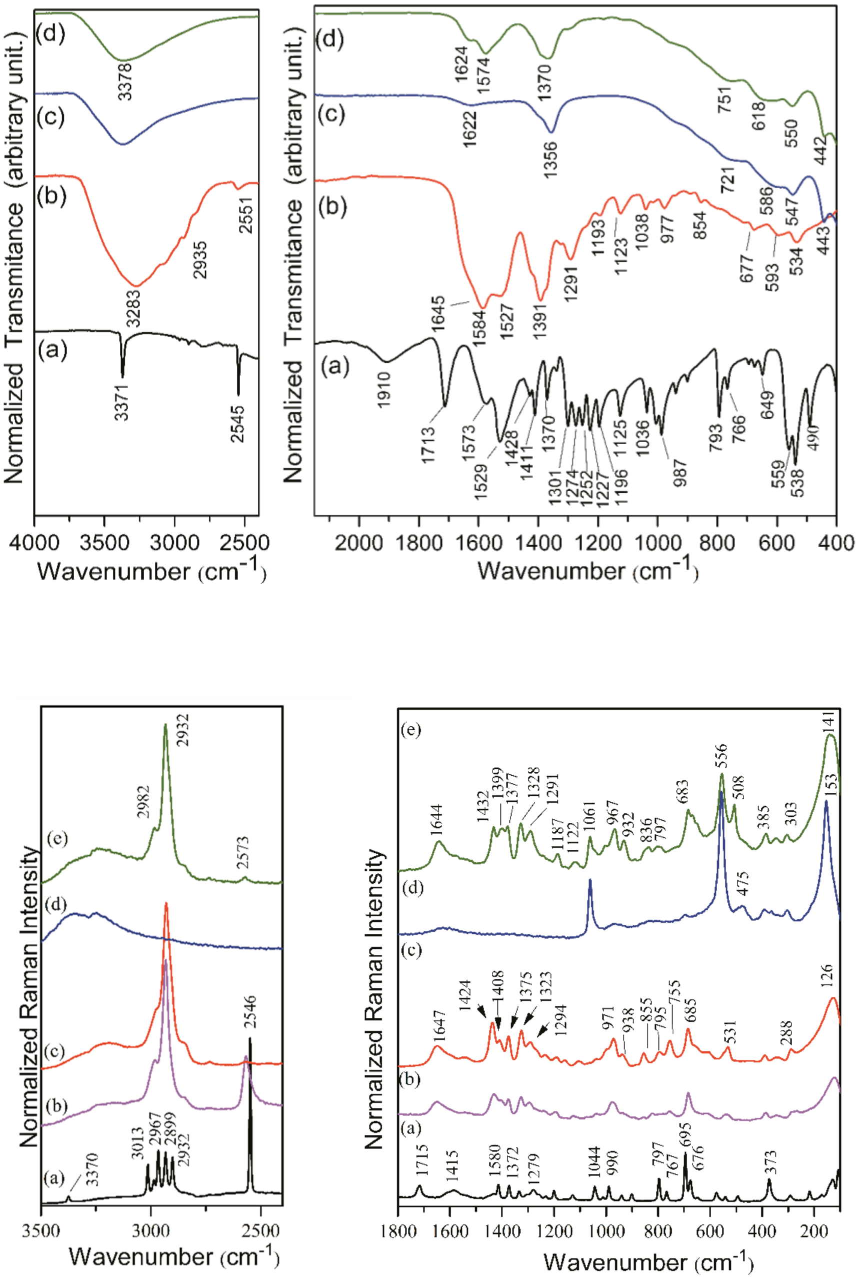


**Figure S10.** FTIR spectra (top) of (a) NAC, (b) NAC-pH=9.5, (c) $Mg_2Al$-Cl and (d) $Mg_2Al$-NAC, and FT-Raman spectra (down) of (a) NAC, (b) NAC-pH=9.5, (c) NAC-pH=11.0, (d) $Mg_2Al$-Cl and (e) $Mg_2Al$-NAC.

## 9. $^{13}$C-CPMAS NMR spectra of NAC and $Mg_2Al$-NAC sample

As indicated in **Figure S11**, this work suggests that the resonance peak of the nuclei $^{13}$C in the oxidized NAC (C*) is shifted as follows: C1* from 175.0 to 179.4 ppm, C3* from 28.3 to 36.9 ppm, C5* from 23.3 to 19.9 ppm. For the reduced NAC, C1 peak (177.2 ppm) is located at the same value as that for $Zn_2Al$-NAC55 material (177.8 ppm), but C2 and C3 peaks are distinct due to the *thiol* group oxidation. Additionally, the peak at 170.7 ppm (marked as C# in $Mg_2Al$-NAC) is attributed to the LDH intercalated with carbonate ion [3] due to atmospheric $CO_2$ contamination.

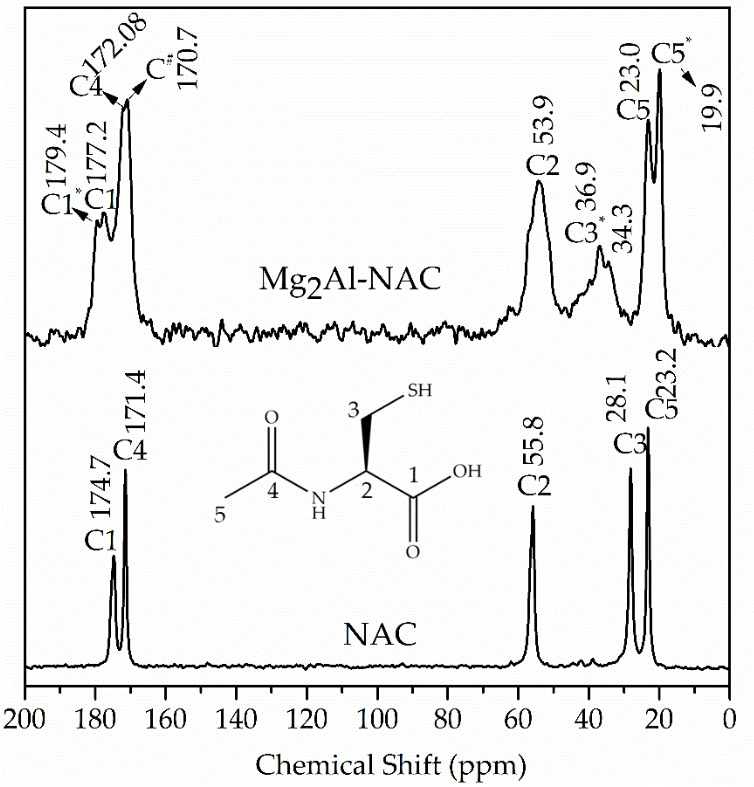


**Figure S11.** $^{13}$C-CPMAS NMR spectra of NAC and $Mg_2Al$-NAC samples.

## 10. Kinetic models applied to the $Zn_2Al$-NAC55 sample

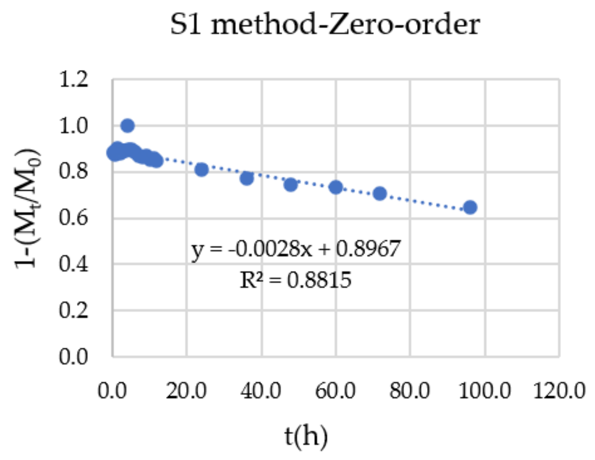


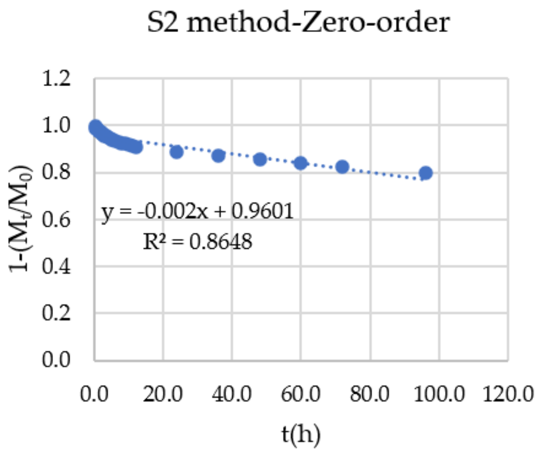


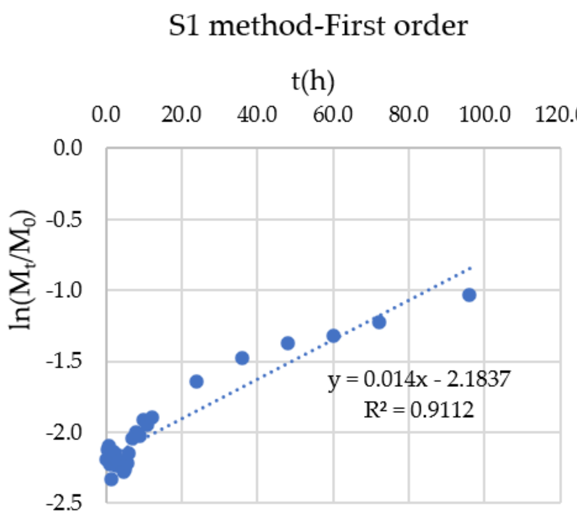


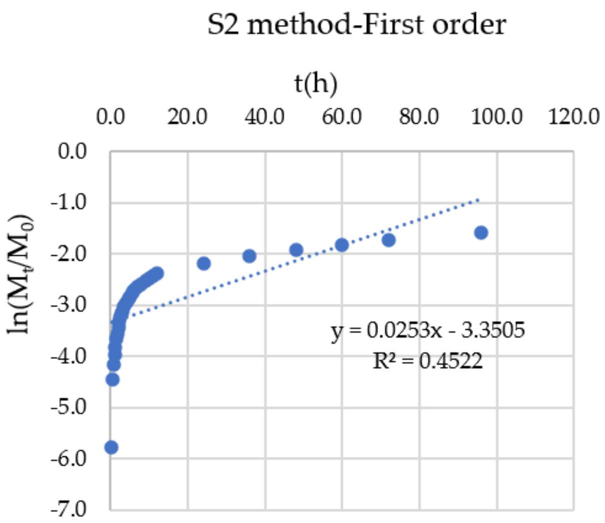


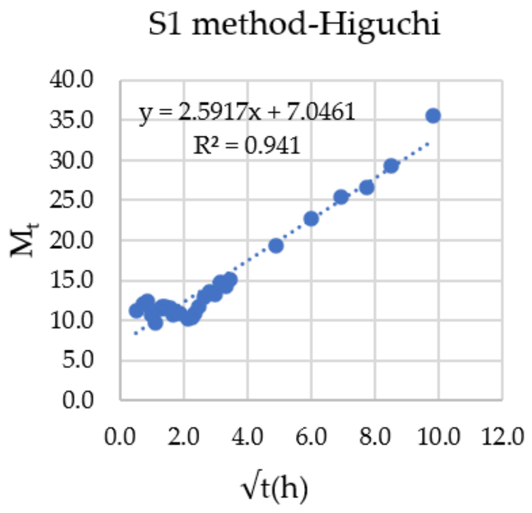


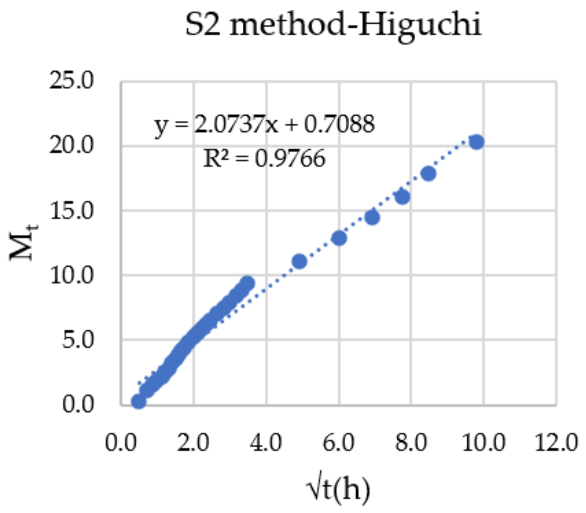

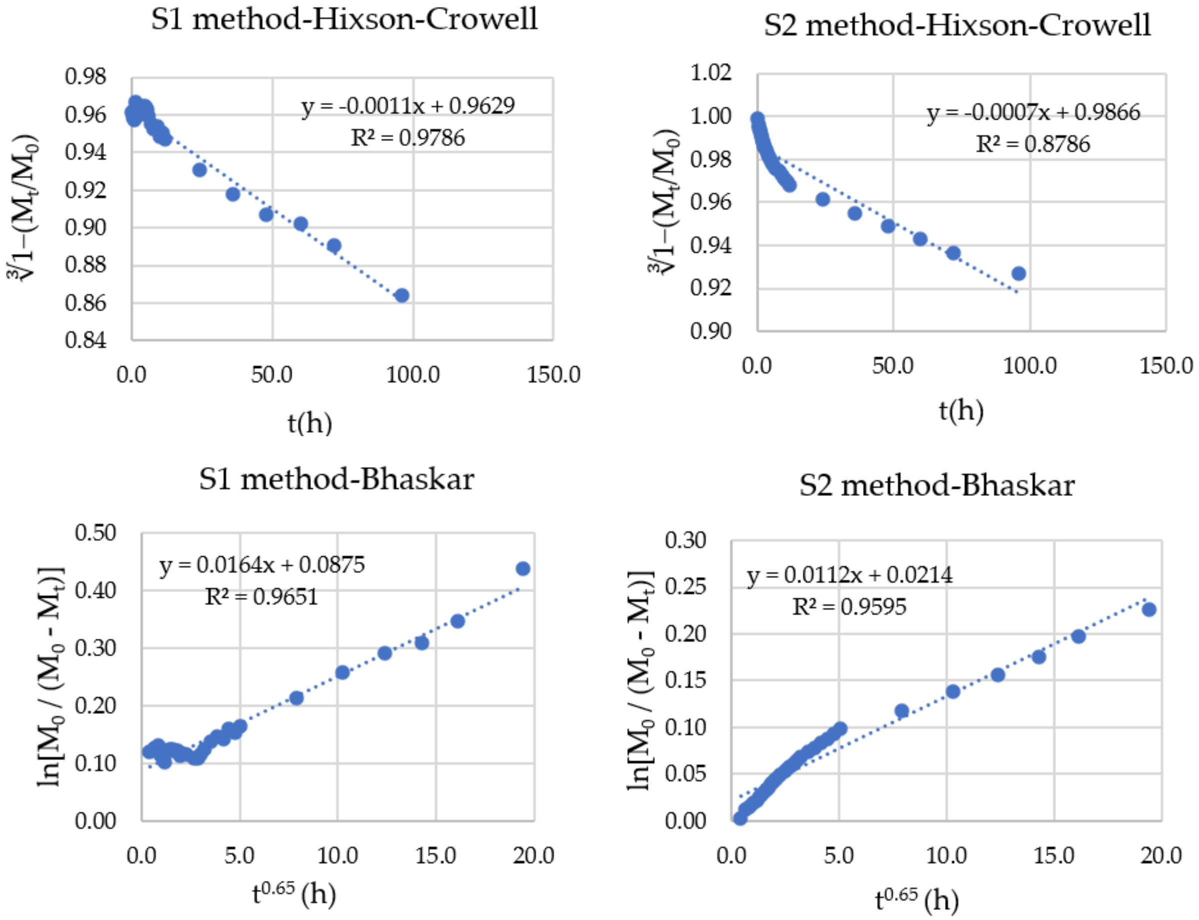


**Figure S12.** Kinetic models applied to the $Zn_2Al$-NAC55 sample, $M_t$ is the amount of NAC released at time t and $M_0$ is the initial amount of NAC.

## REFERENCES


1. Kumar, S.S.; Nangia, A. A New Conformational Polymorph of N-Acetyl-l-Cysteine. The Role of S–H⋯O and C–H⋯O Interactions. *CrystEngComm* **2013**, *15*, 6498, doi:10.1039/c3ce40879e.
2. Cunha, V.R.R.; de Souza, R.B.; da Fonseca Martins, A.M.C.R.P.; Koh, I.H.J.; Constantino, V.R.L. Accessing the Biocompatibility of Layered Double Hydroxide by Intramuscular Implantation: Histological and Microcirculation Evaluation. *Sci. Rep.* **2016**, *6*, 30547, doi:10.1038/srep30547.
3. Olszówka, J.E.; Karcz, R.; Kry, J.; Napruszewska, B.D.; Sulikowski, B.; Socha, R.P.; Gawe, A. New Insight into the Preferred Valency of Interlayer Anions in Hydrotalcite- like Compounds : The effect of Mg / Al Ratio. *Appl. Clay Sci.* **2018**, *155*, 84–94, doi:10.1016/j.clay.2018.01.013.
